# Feasibility of critical care ergometry: Exercise data of patients on mechanical ventilation analyzed as nine‐panel plots

**DOI:** 10.14814/phy2.15213

**Published:** 2022-03-13

**Authors:** Huub L. A. van den Oever, Mert Kök, Aloys Oosterwegel, Emily Klooster, Siebrand Zoethout, Erwin Ruessink, Bas Langeveld

**Affiliations:** ^1^ Intensive Care Unit Deventer Hospital Deventer Netherlands; ^2^ Department of Rehabilitation Deventer Hospital Deventer Netherlands; ^3^ Department of Pulmonology Deventer Hospital Deventer Netherlands

**Keywords:** circulation, critical care, exercise physiology, mechanical ventilation, oxygen consumption

## Abstract

Nine‐panel plots are standard displays of cardiopulmonary exercise data, used in cardiac and pulmonary medicine to investigate the nature of exercise limitation. We explored whether this approach could be used to analyze the data of critically ill patients on mechanical ventilation, capable of exercising actively. Patients followed an incremental exercise protocol using a bedside cycle ergometer. Respiratory gases were analyzed using indirect calorimetry, and blood gases were sampled from arterial catheters. Data of seven patients were combined into nine‐panel plots. Systematic analysis clarified the nature of exercise limitation in six cases. Resting metabolic rate was increased in all patients, with a median oxygen uptake (${\dot{V}O}_{2}$) of 5.52 (IQR 4.29–6.31) ml/kg/min. Unloaded cycling increased the ${\dot{V}O}_{2}$ by 19.8% to 6.61 (IQR 5.99–7.08) ml/kg/min. Adding load to the ergometer increased the ${\dot{V}O}_{2}$ by another 20.0% to reach ${\dot{V}O}_{2\text{peak}}$ at a median of 7.14 (IQR 6.67–10.75) ml/kg/min, corresponding to a median extrinsic workload of 7 W. This was accompanied by increased CO_2_ production, respiratory minute volume, heart rate, and oxygen pulse. Three patients increased their ${\dot{V}O}_{2}$ to >40% of predicted ${\dot{V}O}_{2\max}$, two patients passed the anaerobic threshold. Dead space ventilation was 44%, decreasing to 42% and accompanied by lower ventilatory equivalents during exercise. Exercise produced no net change in alveolo‐arterial PO_2_ difference. We concluded that diagnostic ergometry in mechanically ventilated patients was feasible. Analysis of the data as nine‐panel plots provided insight into individual limitations to exercise.

## Graphical abstract

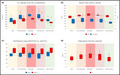

**New & Noteworthy**

In this study, a standardized method for analyzing the data of diagnostic cardiopulmonary exercise testing was applied to an unlikely cohort: Patients on a mechanical ventilator, mobilizing actively on an in‐bed cycle ergometer, while recovering from critical illness. Nine‐panel plot analysis quantifies the limitation to exercise and tries to locate it to cardiac, ventilatory, gas exchange, or musculoskeletal dysfunction. Though ICU patients produced only small amounts of external work, a step‐by‐step analysis provided insight in their exercise limitations. Critical care ergometry may be of diagnostic value when patients have difficulty weaning from mechanical ventilation.

## 1 INTRODUCTION

Cardiopulmonary exercise testing (CPET) is used in cardiac and pulmonary medicine to understand, define and evaluate the nature of exercise limitations. Generally, patients visit outpatient clinics to undergo diagnostic CPET.

Clinicians and physiologists have developed guidelines to standardize testing protocols (Radtke et al., 2019) and data analysis (American Thoracic Society and American College of Chest Physicians, 2003; Roca et al., 1997). The nine‐panel plot is a commonly used method to visualize physiological changes during exercise, and to facilitate interpretation (Wasserman et al., 2012).

Critically ill patients on mechanical ventilation suffer from exercise limitations due to muscle weakness and loss of aerobic function (Skjorten et al., 2021; Wischmeyer et al., 2017). Prolonged immobility during intensive care unit admission may contribute to these problems (Heyland et al., 2016). Despite limited evidence for efficacy (Doiron et al., 2018), current guidelines recommend passive and active exercises for ICU patients to avoid immobility (Devlin et al., 2018).

The aim of the present study was to examine the feasibility of critical care ergometry, by applying existing principles of CPET analysis to the exercise data of actively cycling mechanically ventilated patients.

## 2 METHODS

### 2.1 Study design

This was a prospective, observational study, in which we analyzed exercise data of patients admitted to the mixed medical‐surgical 12‐bed ICU of the Deventer Hospital. The study protocol was submitted to the Ethical Review Board of Isala Clinics, Zwolle, Netherlands, as an observational study during physiotherapy treatment in which increasing bed‐based cycling was provided and cardiorespiratory parameters were measured. The Ethical Review Board approved the study protocol.

### 2.2 Inclusion criteria

Patients older than 18 years with an endotracheal or tracheostomy tube in place, capable of active cycling on an in‐bed cycle ergometer, were eligible for inclusion. Patients who had contra‐indications to physical exercise (Sommers et al., 2015) did not participate. Adverse events were graded according to the Common Terminology Criteria for Adverse Events (National Cancer Institute, 2020).

### 2.3 Data extraction and measurement

We extracted biometric and clinical data from the electronic medical records. If patients had COPD, forced expiratory volume in one second (FEV_1_) was taken from their latest spirometry exam.

Throughout the exercise protocol, gas exchange was analyzed by the Quark metabolic monitor (COSMED, Italy). After calibration, the calorimeter was prepared for measuring resting energy expenditure (REE). The Flow‐REE flowmeter was placed between the tube and the Y‐piece of the ventilatory circuit. During exercise, the Quark recorded the following parameters breath‐by‐breath: Oxygen uptake (${\dot{V}O}_{2}$), carbon dioxide output (${\dot{V}\text{CO}}_{2}$), expiratory minute ventilation ($\dot{V}E$), tidal volume (V_T_), respiratory rate, mixed‐expired pressures of CO_2_ (P_E_CO_2_) and O_2_ (P_E_O_2_), end‐tidal pressures of CO_2_ (P_ET_CO_2_) and O_2_ (P_ET_O_2_), and inspiratory (T_I_) and expiratory times (T_E_). Heart rate (HR) and peripheral oxygen saturation (SpO_2_) were monitored using an integrated pulse oximeter.

We continuously monitored intra‐arterial blood pressure and collected blood samples from an arterial line before and directly after exercise for blood gas and lactate analysis. The delivered performance in Watt (W) was extracted from the ergometer. These data, together with the data from the metabolic monitor were exported to Microsoft Excel for analysis.

### 2.4 Exercise protocol

Patients were positioned in a semi‐recumbent position with their legs placed in a motorized cycling ergometer (MOTOmed Letto2, Reck, Germany) or nonmotorized cycling ergometer (Lode, Groningen, Netherlands). When the MOTOmed was used, the exercise protocol consisted of 5 min rest, followed by 1 min of passive cycling at 20 revolutions per minute (RPM) and 1 or 2 min of unloaded cycling. Thereafter, the resistance was increased with one step every minute. Resistance was decreased if 20 rotations per minute (RPM) were not maintained. The Lode ergometer was mounted on a frame to fit over the foot end of the bed. This device measures work output independent of cycling frequency. When the Lode was used, passive cycling was skipped and the resistance increased stepwise by 2 W every minute. The exercise stopped when patient safety was at risk, or when the patient was unable to continue. After exercise, a 5‐min recovery phase was observed, during which the measurements were continued.

### 2.5 Offline analysis

In MS Excel, all measurements were averaged as recommended in the ATS/ACCP guideline (American Thoracic Society and American College of Chest Physicians, 2003) by applying a moving average over 30–60 s to the raw breath‐by‐breath gas analysis and hemodynamic data. Composite parameters (ratios and differences used in the analysis) were calculated automatically by Cosmed software, or manually using MS Excel (see below). The resulting values were presented as nine‐panel plots according to the fifth edition of Wasserman's textbook (Wasserman et al., 2012). The values of 40% of ${\dot{V}O}_{2\max}$ in panel 1, and maximal predicted HR in panel 2 were indicated with horizontal lines. Where applicable, AT was indicated with a vertical line. Blood pressure was omitted from panel 5, because the values could not be imported synchronously from the bedside monitor. For panels 3, 6, and 9, which did not have time on the horizontal axis, only measurements during exercise were used. Three clinicians (H.v.d.O., B.L., and M.K.) generated a test report, based on the structured interpretation standard of Wasserman, but also including subjective pattern recognition. We used nonparametric statistics for descriptive purposes.

### 2.6 Calculated parameters and normal values

#### 2.6.1 Ideal body weight

Ideal body weight (kg) was calculated according to Wasserman et al. (2012):

Height in cm.

Male:

Ideal bodyweight=0.79×height‐60.7

Female:

Ideal bodyweight=0.65×height‐42.8

#### 2.6.2 Resting metabolic rate (RMR)

The RMR (ml/min/kg, actual body weight) is the oxygen consumption (${\dot{V}O}_{2}$) at rest, averaged over a certain amount of time, for example, 3 min in our study. RMR was compared to published normal values (mean ± SD) (Kwan et al., 2004).

$$\begin{array}{c} \text{Male}<65\;\text{years 3}.03\pm 0.33,\;\text{male}\geq 65\;\text{years}\;\\ 2.84\pm 0.34 \end{array}$$

$$\begin{array}{c} \text{Female}<65\;\text{years 3}.32\pm 0.46,\;\text{female}\geq 65\;\text{years}\;\\ 2.82\pm 0.37 \end{array}$$

RMR was considered abnormal if it exceeded mean ± 2 × SD.

#### 2.6.3 Predicted maximum oxygen consumption (${\dot{V}O}_{2\max}$)

${\dot{V}O}_{2\max}$ (L/min) was calculated according to Wasserman et al. (2012):

Body height in cm, age in years.

Male:

If actual body weight ≥ ideal body weight:

$$\begin{array}{c} {\dot{V}O}_{2\max}=0.0337\;\times \;\text{height}\;‐\;0.000165\times \;\text{age}\;\times \;\text{height}\;\\ \;‐\;1.963\;+\;0.006\;\times \;\text{weight}\;\left(\text{actual}\;‐\;\text{ideal}\right) \end{array}$$

If actual weight < ideal weight:

$$\begin{array}{c} {\dot{V}O}_{2\max}=0.0337\;\times \;\text{height}\;‐\;0.000165\;\times \;\text{age}\;\times \;\text{height}\;\\ \;‐\;1.963\;+\;0.014\;\times \;\text{weight}\;\left(\text{actual}\;‐\;\text{ideal}\right) \end{array}$$

Female:

$$\begin{array}{c} {\dot{V}O}_{2\max}=0.001\;\times \;\text{height}\;\times (14.783\;‐\;0\text{.11}\;\times \;\text{age)}\;\\ \;+\;0.006\;\times \;\text{weight}\;\left(\text{actual}\;‐\;\text{ideal}\right) \end{array}$$

Age of 30 years was used for adults <30 years. ${\dot{V}O}_{2\text{peak}}$ > 84% of predicted ${\dot{V}O}_{2\max}$ was considered normal (American Thoracic Society and American College of Chest Physicians, 2003).

#### 2.6.4 Metabolic equivalent for task (MET)

Metabolic equivalent for task is the ratio of ${\dot{V}O}_{2}$ (ml/min) during a specific task over RMR, which is ${\dot{V}O}_{2}$ at rest (Heydenreich et al., 2019).

$$\text{MET}={\dot{V}O}_{2\text{task}}/\text{RMR}$$

#### 2.6.5 Oxygen cost of work

During loaded exercise, oxygen consumption increases linearly with the external workload. An increase in ${\dot{V}O}_{2}$ of 8.5–11.5 ml/min per W external exercise was considered normal (Hansen et al., 1988). When plotted in the same graph with a 10‐fold difference in scale (as in panel 1), the increases of ${\dot{V}O}_{2}$ and work rate should have similar slopes.

#### 2.6.6 Recovery of ${\dot{V}O}_{2}$ after exercise

When at the end of an exercise test, the exercise is reduced to unloaded cycling, the oxygen consumption decreases to baseline within minutes. The ${\dot{V}O}_{2}$ recovery half‐time is determined graphically. A ${\dot{V}O}_{2}$ recovery half‐time shorter than 100 s was considered normal (Klainman et al., 2008).

#### 2.6.7 Predicted maximum heart rate

In healthy individuals, at maximum exercise ${\dot{V}O}_{2}$ ceases to increase due to circulatory limitation to O_2_ transport. Therefore, in a maximal exercise test, participants HR should approximate the predicted maximum HR. It is normal for maximum heart rate to decrease with age (Karvonen et al., 1957).

$$\begin{array}{c} \text{Predicted maximum HR}\;(\mathrm{bpm})\;=220\;‐\;\text{age}\;\left(\text{years}\right) \end{array}$$

#### 2.6.8 Heart rate reserve (HRR)

Heart rate reserve (in bpm) was calculated as:

$$\begin{array}{c} \text{HRR}\;=\;\text{predicted maximum HR}\;\;\\ \;‐\;\text{observed peak HR}\; \end{array}$$

When maximum exercise is delivered, heart rate reserve should be <15 bpm (American Thoracic Society and American College of Chest Physicians, 2003; Wasserman et al., 2012).

#### 2.6.9 O_2_‐pulse

The ratio of oxygen consumption to heart rate, or O_2_‐pulse, expresses the volume of oxygen ejected from the ventricles with each cardiac contraction.

$$O_{2}\;p\text{ulse}\;=\dot{V}O_{2}/\text{HR}\;\left(\text{ml}/\text{beat}\right)$$

#### 2.6.10 Predicted maximum O_2_‐pulse

If maximum exercise is delivered, O_2_‐pulse (in ml/heart beat) should be >80% of predicted maximum O_2_‐pulse.

$$\begin{array}{c} \mathrm{Predicted}\;\mathrm{maximum}\;O_{2}\;\mathrm{pulse}\;=\\ \mathrm{predicted}\;\dot{V}O_{2\max}/\mathrm{predicted}\;\mathrm{maximum}\;\mathrm{HR} \end{array}$$

(American Thoracic Society and American College of Chest Physicians, 2003; Wasserman et al., 2012).

#### 2.6.11 Ventilatory equivalent

The ventilatory equivalent for CO_2_ (eqCO_2_) represents the amount of ventilation required for the removal of each liter of carbon dioxide, and inversely reflects ventilatory efficiency. During exercise, the eqCO_2_ tends to decrease, mostly due to reduced dead space ventilation, which improves the efficiency of ventilation. Values <34 at the AT and <36 at peak exercise were considered normal (American Thoracic Society and American College of Chest Physicians, 2003; Wasserman et al., 2012).

$${\mathrm{eqCO}}_{2}=\dot{V}E/\dot{V}{\mathrm{CO}}_{2}$$

The ventilatory equivalent for O_2_ (eqO_2_) represents the amount of ventilation required for the consumption of each liter of oxygen, and reflects ventilatory efficiency for oxygen. During exercise, the eqO_2_ tends to decrease, mostly due to reduced dead space ventilation, which improves the efficiency of ventilation. Above the anaerobic threshold, when ventilation is increased due to lactate production, the eqO_2_ starts to increase. Values between 23 and 28 were considered normal (American Thoracic Society and American College of Chest Physicians, 2003; Wasserman et al., 2012).

$${\mathrm{eqCO}}_{2}=\dot{V}E/\dot{V}O_{2}$$

#### 2.6.12 Maximum voluntary ventilation (MVV)

MVV is the largest amount of air that a person can inhale and then exhale during a time interval with maximal voluntary effort. The MVV is primarily determined by the time required for expiration.

An estimation of MVV is based on the forced expiratory volume (FEV_1_), the maximum amount of air a person can exhale in 1 s. As the expiration time occupies approximately 2/3 of the respiratory cycle, it was assumed that 40 s of each minute are occupied by expiration (Wasserman et al., 2012).

$$\text{MVV}\;=40\;\times {\text{FEV}}_{1}$$

#### 2.6.13 Breathing reserve (BR)

BR is the difference between the observed minute ventilation at peak exercise and the (theoretical) maximum voluntary ventilation. Healthy persons have a large breathing reserve (>11 L/min for healthy males), even at peak exercise, but in obstructive lung disease $\dot{V}E$ can become the limit to exercise (Wasserman et al., 2012).

$$\begin{array}{c} \text{BR}\;\left(L/\min\right)=\;\text{MVV−}\dot{V}E_{\text{peak}}\; \end{array}$$

#### 2.6.14 Ideal alveolar partial pressure of O_2_ (P_A_O_2_)

At present no method exists to directly measure the PO_2_ in alveoli. A theoretical approach to calculate P_A_O_2_ uses values available from blood gas analysis and calorimetry (Wasserman et al., 2012).

$$\begin{array}{c} P_{A}O_{2}={\;F}_{i}O_{2}\times \left(P_{B}-\;{\;P}_{\text{H2O}}\right)\;-\;\left(\left(P_{A}{\text{CO}}_{2}/\;\text{RER}\right)\;\right.\\ \;\left.\times \;\left(1-{\;F}_{i}O_{2}\times \;\left(1-\;\text{RER}\right)\right)\right) \end{array}$$

where F_i_O_2_ is the fraction of inspired oxygen; P_B_ is the barometric pressure in kPa, measured during calibration by the Quark; and P_H2O_ is the partial pressure of water vapor (6.27 kPa at BTPS). We used P_a_CO_2_ as a substitute for P_A_CO_2_. RER was measured by the metabolic analyzer.

#### 2.6.15 Alveolo‐arterial PO_2_ difference (P_A‐a_O_2_)

The difference between the ideal alveolar PO_2_ and the arterial PO_2_ was calculated before and directly after exercise. A value <4.66 kPa at maximal exercise was considered normal (American Thoracic Society and American College of Chest Physicians, 2003).

#### 2.6.16 Fractional dead space (V_D_/V_T_)

A unitless value, calculated using the modified Bohr equation in which P_a_CO_2_ replaces P_A_CO_2_ (Wasserman et al., 2012).

$$\begin{array}{c} V_{D}/V_{T}=\;\left(P_{a}{\text{CO}}_{2}‐P_{E}{\text{CO}}_{2}\right)\;/\;P_{a}{\text{CO}}_{2} \end{array}$$

in which P_E_CO2 was calculated by the Quark using volumetric capnography. V_D_/V_T_ was calculated before and directly after exercise. A value <0.4 at rest and <0.28 (for participants ≤40 years), or <0.30 (for participants >40 years) at maximal exercise was considered normal (American Thoracic Society and American College of Chest Physicians, 2003).

#### 2.6.17 P(_a‐ET_)CO_2_

Due to mixing of alveolar gases with dead space gases in the airways, the alveolar PCO_2_ (close to the arterial PCO_2_) is always higher than the end‐tidal PCO_2_ (measured at the mouth‐piece). Therefore, large gap between arterial PCO_2_ and end‐tidal PCO_2_ reflects increased physiological dead space. Normal values at rest were −0.04 ± 0.39 kPa, and at peak exercise −0.55 ± 0.43 kPa (Hansen et al., 1984).

#### 2.6.18 P_(ET‐E)_CO_2_

A large difference between end‐tidal PCO_2_ and mix‐expired PCO_2_ occurs when there is a slope in the expiratory phase of the capnogram. This is thought to represent inhomogeneous expiration, as is encountered in obstructive lung disease. A value of <1.7 kPa at peak exercise was considered normal.

## 3 RESULTS

### 3.1 Patient enrolment

We included seven patients to the incremental exercise protocol, all recovering from a systemic inflammatory syndrome. Two patients were on a low dose of noradrenalin and/or milrinone at the time of testing. Table 1 shows baseline characteristics of the participants. We created an individual test report for each patient. The complete set of nine‐panel exercise plots and the accompanying ergometry reports are published online (Supporting Material). The individual plots and reports are referred to with a hash tag (#) followed by the patient number.

**TABLE 1:** Baseline characteristics of the participants

Characteristic	(*n* = 7)
Sex, male, *n* (%)	4 (57.1%)
Age, years	71 (59–75.5)
Charlson comorbidity index	5 (2–5)
Admission diagnosis, *n* (%)	
Pneumosepsis	5 (71.4%)
Pancreatitis	1 (14.3%)
Postanoxic encephalopathy	1 (14.3%)
APACHE IV	92 (82–108)
Relevant medication, *n* (%)	
Inotropic	1 (14.3%)
Vasopressor	2 (28.6%)
Beta‐blocker	1 (14.3%)
Antiarrhythmic	1 (14.3%)
ICU stay to inclusion (days)	7 (4.5–16.5)
Mechanical ventilation to inclusion (days)	7 (3–15.5)
Total duration of mechanical ventilation (days)	11 (3–28)
Type of intubation	
Endotracheal, *n* (%)	4 (57.1%)
Tracheostomy, *n* (%)	3 (42.9%)
Ventilation mode	
Pressure support, *n* (%)	6 (85.7%)
Volume control, *n* (%)	1 (14.3%)
Driving pressure (above PEEP; cm H_2_O)	8 (7–10)
PEEP (cm H_2_O)	8 (7–8)
F_i_O_2_ (%)	35 (25–35)

Note

Values are median and interquartile range unless specified otherwise.

Abbreviations: APACHE IV, acute physiology and chronic health evaluation IV; FiO_2_, fraction of inspired oxygen; ICU, intensive care unit; PEEP, positive end‐expiratory pressure.

### 3.2 General observations

All patients had a Richmond Agitation and Sedation Score of 0, indicating that they were awake and cooperative. Patients could not be instructed for pretest spirometry (to determine FEV_1_ and vital capacity). Two patients exercised on the Lode ergometer and five on the MOTOmed. Average exercise duration was 8 min. The pedaling rate was maintained at 20–30/min by physiotherapists’ instructions. Some patients could not keep the pedaling rate constant, and in an attempt to maintain it, the resistance was lowered, leading to nonlinear workload increments in some instances (#5 and #6). Most patients stopped due to leg fatigue; one test was stopped because of systolic hypertension of 230 mmHg (#3), which stabilized during the recovery period (graded as a severe adverse event). Patient #1, with a history of intermittent atrial fibrillation, developed atrial fibrillation during exercise (moderate adverse event).

### 3.3 Panel 1: O_2_ uptake (${\dot{V}O}_{2}$), CO_2_ production (${\dot{V}\text{CO}}_{2}$), and workload versus time

**FIGURE 1:**
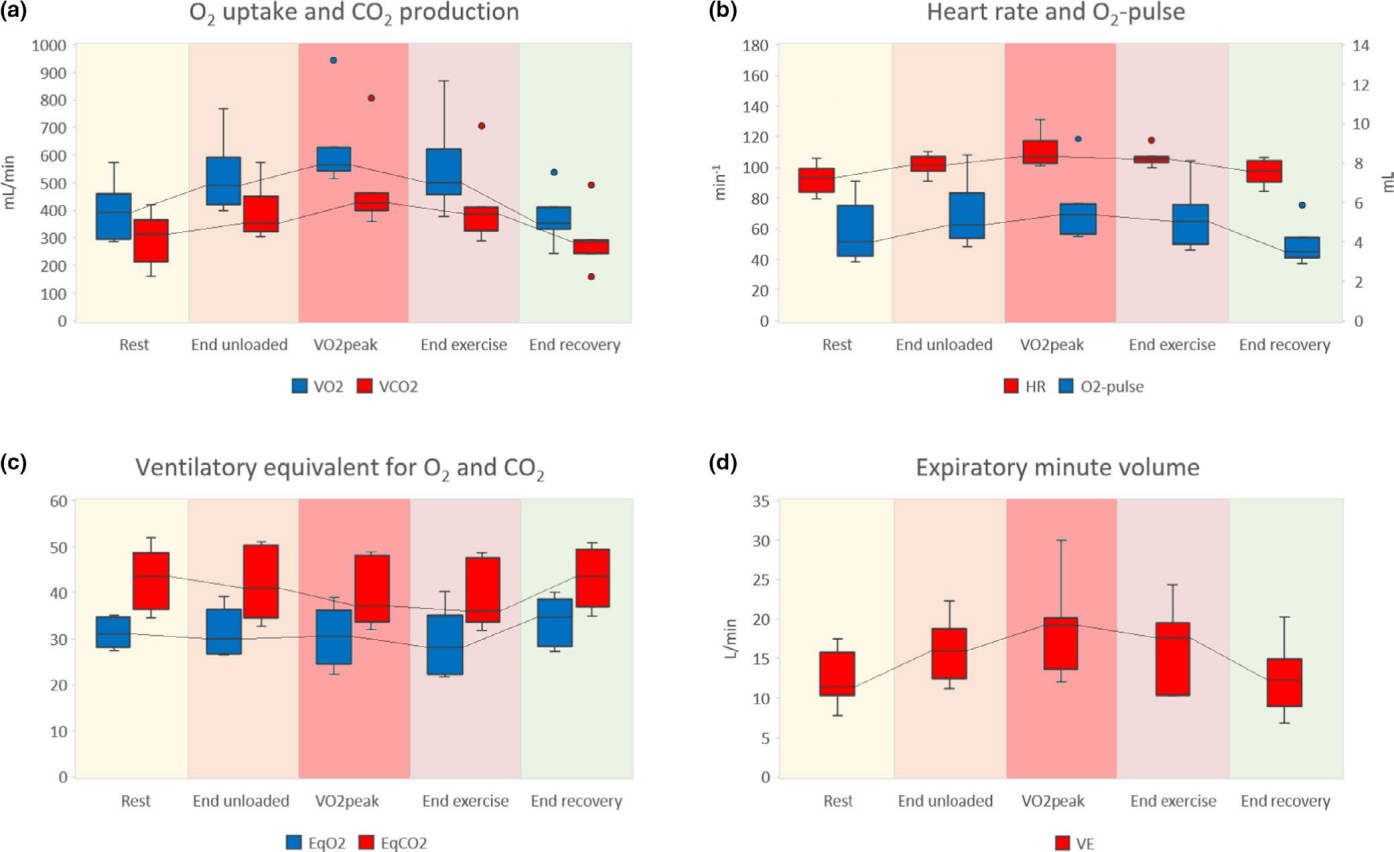
Respiratory gas analysis of seven patients during five phases of exercise. Panels (a–d) correspond to panels 1, 2, 4, and 5 of the nine‐panel plots proposed by Wasserman (5th Edition, 2012), respectively. Panel (a) shows O_2_ uptake (${\dot{V}O}_{2}$) and CO_2_ production (${\dot{V}\text{CO}}_{2}$), panel (b) shows heart rate (HR) and O_2_‐pulse, panel (c) shows the ventilatory equivalent for O_2_ (EqO_2_) and CO_2_ (EqCO_2_), and panel (d) shows the expiratory minute volume ($\dot{V}E$).${\dot{V}O}_{2\text{peak}}$ did not always coincide with the end of exercise

The resting metabolic rate was elevated in all patients; median ${\dot{V}O}_{2}$ at rest was 5.52 (IQR 4.29–6.31) ml/kg/min. During unloaded exercise, ${\dot{V}O}_{2}$ increased by 19.8% to 6.61 (IQR 5.99–7.08) ml/kg/min. During cycling with incremental load increase, ${\dot{V}O}_{2}$ increased by another 20.0% to reach ${\dot{V}O}_{2\text{peak}}$ at a median of 7.14 (IQR 6.67–10.75) ml/kg/min, which did not always coincide with the end of exercise. None of the patients reached the predicted ${\dot{V}O}_{2\max}$. Three participants (#2, 3, and 7) surpassed 40% of predicted ${\dot{V}O}_{2\max}$ (median ${\dot{V}O}_{2\text{peak}}$/${\dot{V}O}_{2\max}$ ratio: 34.3%; IQR 29.1–45.3%). Median ${\dot{V}O}_{2}$ and ${\dot{V}\text{CO}}_{2}$ with IQRs during different test phases are illustrated in Figure 1, panel a.

Metabolic equivalents (METs) varied from 1.21 to 1.89, with a median of 1.55 (IQR 1.47–1.69). The median external workload was 7 W (IQR 5.5–10 W). The difference between median ${\dot{V}O}_{2\text{peak}}$ and ${\dot{V}O}_{2}$ at the end of unloaded exercise was 73 ml/min, resulting in a Δ${\dot{V}O}_{2}$/Δ workload of 10.4 ml/min/W. Three patients (#1, 4, 7) demonstrated a normal decrease of ${\dot{V}O}_{2}$ during recovery (median ${\dot{V}O}_{2}$ half‐time: 83 s; IQR 44–116 s). ${\dot{V}O}_{2}$ half‐time could not be assessed in one patient (#6).

### 3.4 Panel 2: heart rate and O_2_‐pulse versus time

The participants had a median resting HR of 93 (IQR 88–98) bpm, which increased by 8% to 101 (IQR 100–105) bpm during unloaded cycling, and by another 7% to a maximum of 109 (IQR 108–115) bpm at peak exercise. Median heart rate reserve at peak exercise was 42 (IQR 34–53) bpm. In one patient (#1), the maximum heart rate during the test approached the predicted maximum heart rate (heart rate reserve <15 bpm).

The O_2_‐pulse was 4.02 (IQR 3.48–5.20) ml/heartbeat at rest, increasing by 21% to 4.86 (IQR 4.33–5.70) ml during unloaded cycling, and by another 11% to 5.39 (IQR 4.83–5.70) ml/heartbeat at peak exercise. All patients remained far from their predicted O_2_‐pulse at maximum exercise. Median HR and O_2_‐pulse during different test phases are illustrated in Figure 1, panel b. One patient (#1) with a history of intermittent atrial fibrillation (AF), developed AF during exercise. At that point, he reached his circulatory limit (heart rate reserve of 10 bpm) and showed an abrupt drop in O_2_‐pulse. Two other participants (#2, 3) showed a mild decrease in O_2_‐pulse toward the end of exercise, without electrocardiographic changes.

### 3.5 Panel 3: carbon dioxide removal (${\dot{V}\text{CO}}_{2}$) and heart rate versus oxygen uptake (${\dot{V}O}_{2}$)

${\dot{V}\text{CO}}_{2}$ increased linearly with ${\dot{V}O}_{2}$ until the end of exercise in most patients. According to the V‐slope and ventilatory equivalent methods (American Thoracic Society and American College of Chest Physicians, 2003), two participants (#2, 3) passed the anaerobic threshold (AT) at 39.0% and 39.1% of predicted ${\dot{V}O}_{2\max}$, respectively. This was accompanied by a rise in serum lactate (from 2.4 to 3.1 mmol/L and from 0.9 to 3.1 mmol/L, respectively).

A linear increase of HR in relation to ${\dot{V}O}_{2}$ was noted in five participants (#2–6); in patient #1 the relation was obscured by the onset of AF during exercise. In three (#2, 4, and 5), the HR/${\dot{V}O}_{2}$ slope was steeper than predicted. Patient #2 was on inotropic support and #5 had a high HR due to AF; no abnormality was identified in the other patients.

### 3.6 Panel 4: ventilatory equivalents for O_2_ and CO_2_ (EqO_2_ and EqCO_2_) versus time

Four participants (#4–7) demonstrated improved ventilatory efficiency for oxygen uptake during exercise, reflected by decreasing EqO_2_. At rest, median EqO_2_ was 31.1 (IQR 28.7–34.7), decreasing to 28.1 (IQR 24.9–33.3) at the end of exercise. Median eqO_2_ and eqCO_2_ with IQRs during different test phases are illustrated in Figure 1, panel c.

Similarly, all except for one patient (#2), showed improved ventilatory efficiency for carbon dioxide removal during exercise, reflected by a decreasing trend of EqCO_2_. In rest, median EqCO_2_ was 43.5 (IQR 39.2–48.4), decreasing to 35.9 (IQR 34.3–45.2) at the end of exercise.

### 3.7 Panel 5: expiratory minute volume ($\dot{V}E$) versus time

At rest, median $\dot{V}E$ was 11.4 (IQR 10.5–15.4) L/min, which increased by 40% to 16 (IQR 13.5–17.3) during unloaded cycling, and further increased by 20% to 19.2 (IQR 14.3–20.1) at ${\dot{V}O}_{2\text{peak}}$ (Figure 1, panel d). Due to the absence of spirometry data, $\dot{V}E$ could not be interpreted in relation to the predicted maximum voluntary ventilation.

### 3.8 Panel 6: $\dot{V}E$ versus ${\dot{V}\text{CO}}_{2}$

Our cohort had a median $\dot{V}E$/${\dot{V}\text{CO}}_{2}$ slope of 29.3 (IQR 28.3–40.4). Three (#3, 4, 6) had values between 38.1 and 44.9, exceeding the upper limit of predicted normal values. An extremely low slope of 17.3 in patient #7 was attributed to insufficient ability to increase $\dot{V}E$ due to controlled ventilation. In this patient, the EqCO_2_ at the AT was 33.7 (normal). None of the patients reached the respiratory compensation point.

### 3.9 Panel 7: partial pressures for O_2_ and CO_2_ versus time

An overview of the values extracted from this plot is presented in Table 2. P_E_O_2_ and P_ET_O_2_ remained unchanged in five patients during exercise. In two patients (#2, 7), a sudden decrease was observed, coinciding with a change in F_i_O_2_ on the mechanical ventilator, and therefore considered an artifact. The alveolo‐arterial O_2_‐difference (P_A‐a_O_2_) was normal in two patients (#1, 4) and elevated in the other five. There was no consistent change in P_A‐a_O_2_ during exercise. Excluding the two patients in whom the FiO2 was changed during the test, the P_A‐a_O_2_ increased by a median of 0.3 kPa.

**TABLE 2:** Respiratory values of critically ill, mechanically ventilated patients at rest and at the end of an exercise test

Respiratory parameter	Rest	End exercise
P_E_O_2_ (kPa)	28.7 (20.5–29.9)	23.3 (19.9–27.5)
P_ET_O_2_ (kPa)	25.5 (18.9–28.3)	20.4 (18.3–26.0)
P_E_CO_2_ (kPa)	2.87 (2.69–3.33)	3.33 (2.80–3.67)
P_ET_CO_2_ (kPa)	4.67 (3.84–5.20)	5.07 (4.00–5.40)
P_a_O_2_ (kPa)	12.1 (10.6–13.7)	11.2 (10.2–11.6)
P_a_CO_2_ (kPa)	5.00 (4.70–5.70)	5.60 (4.95–6.20)
P_A_O_2_ (kPa)	23.1 (17.2–26.5)	18.7 (16.2–24.4)
P_A‐a_O_2_ (kPa)	9.95 (4.49–14.6)	7.21 (4.92–13.6)
P_a‐ET_CO_2_ (kPa)	0.58 (0.01–0.93)	0.53 (0.18–0.96)
P_ET‐E_CO_2_ (kPa)	1.46 (1.29–1.71)	1.47 (0.90–1.63)
V_D_/V_T_	0.44 (0.29–0.48)	0.42 (0.31–0.44)

Note

The partial pressures are extracted from panel 7 of the exercise plots.

Abbreviations: P_A‐a_O_2_, alveolar to arterial oxygen gradient; P_a_CO_2_, arterial carbon dioxide pressure; P_a‐ET_CO_2_, arterial to end‐tidal carbon dioxide gradient; P_a_O_2_, arterial oxygen pressure; P_A_O_2_, ideal alveolar oxygen pressure; P_E_CO_2_, mixed expired carbon dioxide pressure; P_E_O_2_, mixed expired oxygen pressure; P_ET_CO_2_, end‐tidal carbon dioxide pressure; P_ET‐E_CO_2_, end‐tidal to mixed expired carbon dioxide gradient; P_ET_O_2_, end‐tidal oxygen pressure; V_D_/V_T_, fractional dead space.

In most patients P_ET_CO_2_ and P_E_CO_2_ remained unchanged. In two (#3, 7), a slight increase was observed, but the difference between P_ET_CO_2_ and P_E_CO_2_ remained unchanged.

The arterial pressure of CO_2_ (P_a_CO_2_) was elevated in patient #7 and reduced in #1. P_a_CO_2_ increased by ≥0.5 kPa (range 0.5–1.0 kPa) in four patients (#3, 5–7) during exercise.

At rest, the difference between P_a_CO_2_ and P_ET_CO_2_ (P_a‐ET_CO_2_, considered a measure for dead space ventilation), was normal in three patients (#1, 4, 6) and remained within normal range at the end of exercise. V_D_/V_T_ at rest was within normal range in the same three patients and remained normal at the end of exercise in two. One patient (#6) demonstrated an increase in V_D_/V_T_ during exercise (0.27 at rest to 0.36 at the end of exercise).

### 3.10 Panel 8: respiratory exchange ratio versus time

Median RER at rest was 0.74 (IQR 0.70–0.80) and increased in four participants (#1, 2, 3, 7) during exercise. In three (#2, 3, 7) this increase was attributed to anaerobic metabolism. Median RER at ${\dot{V}O}_{2\text{peak}}$ was 0.77 (IQR 0.70–0.82).

### 3.11 Panel 9: tidal volume versus expiratory minute volume

Median V_T_ at rest was 0.49 (IQR 0.40–0.59) L, increasing to 0.64 L (IQR: 0.51–0.67 L) at ${\dot{V}O}_{2\text{peak}}$. Due to the absence of spirometric data, V_T_ could not be interpreted in relation to vital and inspiratory capacities.

### 3.12 Individual test conclusions

All patients had reduced exercise tolerance. Analyzing the nine‐panel plots revealed one or more possible causes in six patients. Five participants (#2, 3, 5–7) had an insufficient gas exchange, three (#1–3) had suspected cardiovascular limitations, and one (#3) had a suspected ventilatory restriction. Normal gas exchange and the absence of circulatory and respiratory limitations in patient #4 suggested a restriction elsewhere in the musculoskeletal system.

## 4 DISCUSSION

In the present study, diagnostic exercise testing based on clinical guidelines (American Thoracic Society and American College of Chest Physicians, 2003; European Respiratory Society, 1997; Radtke et al., 2019) was applied in a cohort of patients that had never been approached this way before: Patients recovering from critical illness, who were on mechanical ventilation while mobilizing actively on a cycle ergometer. We used the nine‐panel layout proposed by Wasserman (Wasserman et al., 2012) because it is widely used in exercise labs and is often the standard display on diagnostic CPET equipment. The purpose of this study was to determine whether CPET in mechanically ventilated patients was feasible and to evaluate the diagnostic information it would yield.

Even though these patients stopped cycling well below their predicted ${\dot{V}O}_{2\max}$, many elements of CPET analysis could be applied meaningfully. Analyzing their exercise data as nine‐panel plots enhanced our understanding of their limitations to exercise, and we concluded that “critical care ergometry” was feasible.

### 4.1 Diagnostic value

The purpose of clinical ergometry is to quantify the limitation to exercise and locate it to cardiac, ventilatory, gas exchange, or musculoskeletal dysfunction. By pursuing this analysis systematically, we were able to identify one or more limiting factors in six of seven patients. In the other, a musculoskeletal limitation or lack of cooperation was suspected. Whether this approach has diagnostic value in patients recovering from critical illness remains to be established in further and larger studies.

### 4.2 Resting metabolic rate (RMR)

Panel 1 of the nine‐panel plots simultaneously displayed O_2_‐uptake, CO_2_‐production, and workload. Shaded areas in the graphs illustrated the different phases of exercise (rest, unloaded exercise, progressively loaded exercise, and recovery), and each phase added relevant information. The resting phase showed that the RMR was elevated in all patients, despite the fact that they were no longer in active infection, and had no fever. Although the cause of hypermetabolism is not entirely understood, it is well documented. Ventilator‐dependent patients have a RMR that is approximately 25% elevated above the level predicted by the Harris‐Benedict equation (Faisy et al., 2003). In healthy individuals, RMR lies at approximately 10% of ${\dot{V}O}_{2\max}$ (Wasserman et al., 2012) but our patients started off at a much higher metabolic rate: median RMR was 25.9% of their predicted ${\dot{V}O}_{2\max}$.

### 4.3 Unloaded cycling

The initial rise in VO_2_ at the start of unloaded exercise, which most of our patients typically displayed in panel 1, is attributed to a sudden increase in venous return upon contraction of leg muscles, causing a rise in blood flow to the lungs where oxygen is absorbed and carbon dioxide is removed (Wasserman et al., 1974). Figure 1 shows that in the first minute of exercise, ${\dot{V}\text{CO}}_{2}$ and ${\dot{V}O}_{2}$ went up by 13 and 20%, respectively. Still, the median increase in ${\dot{V}O}_{2}$ of 1.1 ml/kg/min was lower than expected. Unloaded cycling at a rate of 60 rpm normally increases the ${\dot{V}O}_{2}$ by 2.3–2.7 ml/kg/min (Whipp & Wasserman, 1969). This discrepancy may have several reasons. First, the duration of unloaded cycling (one minute) may have been too short to reach a plateau. In clinical exercise testing, 3 min of unloaded cycling is current (American Thoracic Society and American College of Chest Physicians, 2003; Wasserman et al., 2012), allowing time for the cardiovascular and ventilatory systems to adjust to the exercise. In our protocol, ${\dot{V}O}_{2}$, ${\dot{V}\text{CO}}_{2}$_,_ and HR may not yet have reached a plateau after 1 min of unloaded cycling, leading to underestimation of these values. Second, all of our patients pedaled at a rate lower than 60 rpm, resulting in a lower intrinsic work rate and a lower ${\dot{V}O}_{2}$. In future critical care ergometry sessions, we will apply 3 min of unloaded cycling, and an additional blood gas analysis at the end of it.

Remarkably, during unloaded cycling our patients reached 76–89% of their achieved ${\dot{V}O}_{2\text{peak}}$.

For any athlete, exercising at 80% of ${\dot{V}O}_{2\text{peak}}$ would be considered high intensity training (Carnes et al., 2018). This may explain why some patients could not keep their pedaling rate constant at 20–30 rpm, even without added load. Though the optimal dose and duration of exercise for critically ill patients are largely unknown, this does not support the practice of adding extra resistance in daily training sessions.

### 4.4 Peak exercise

The peak level in oxygen uptake (${\dot{V}O}_{2\text{peak}}$) was reached between 23.1 and 55.2% of predicted ${\dot{V}O}_{2\max}$, with a median of 34.3%. In patients with severe heart failure, whose ${\dot{V}O}_{2}$ levels may lie in the same performance range, the percentage of predicted ${\dot{V}O}_{2\max}$ they can achieve is a powerful prognostic factor, with a low percentage predicting poor short‐term prognosis (Stelken et al., 1996). The potential to reverse the underlying disease may make this different in ICU patients, but this requires further research.

In terms of external work, the median power delivery at the peak of the activity, as measured by the ergometer, was 7 W. Some caution is required, because according to the manufacturer power data of the MOTOmed are not validated, and this ergometer was used in five of seven cases. Nevertheless, this low work rate seemed plausible, because it resulted in a normal median Δ${\dot{V}O}_{2}$/Δ workload of 10.4 ml/min/W, and an acceptable linearity between ${\dot{V}O}_{2}$ and workload in most individual graphs.

When ${\dot{V}O}_{2\text{peak}}$ was expressed in terms of metabolic equivalent for tasks, median performance was 1.55 MET, with little variance (IQR 1.47–1.69). Translated to daily life activities, this compares to light office work (Ainsworth et al., 2011). Because MET is ${\dot{V}O}_{2}$ divided by the RMR, it might not be the ideal parameter to express performance in patients with an elevated RMR; doing so may have exacerbated the low performance. However, looking at the entire picture, the capacity for external work was very limited.

The exercise tolerance in our patients was consistent with other CPET studies in ICU patients. In a CPET study with 28 mixed ICU patients the ${\dot{V}O}_{2}$ increased from 291 to 384 ml/min, corresponding to 1.32 MET (Sommers et al., 2019). Another study reported a ${\dot{V}O}_{2}$ increase from 249 to 337 L/min, corresponding to 1.35 MET (Akoumianaki et al., 2017). This illustrates the low level of exercise tolerance at which critically ill patients begin their rehabilitation.

### 4.5 Anaerobic threshold

The analysis of Wasserman panel 3, with additional input from panels 1, 4, and 8, suggested that two patients reached their anaerobic threshold. Not surprisingly, these were the participants reaching the highest percentage of their predicted ${\dot{V}O}_{2\max}$ (>40%). In many situations, the V‐slope method indicates where the AT lies, but when exercise is submaximal, the AT may be hard to determine, because assessing the slope of data points above it may become unreliable when the AT is only marginally exceeded. However, both patients showed a concomitant increase in serum lactate. Further support comes from the fact that the AT occurred around 40% of predicted ${\dot{V}O}_{2\max}$, which is where one would expect the AT in an unfit person. None of the patients reached the ventilatory compensation point.

To our knowledge, it has not been documented before that ventilated patients recovering from critical illness perform exercise above the AT, and it occurred at a surprisingly low work rate. With solid research in this area lacking, thought should be given to whether exercise levels around or above the AT are desirable for this type of patient. We hypothesize that improved understanding of the physiology may lead to better and individually adjusted training programs.

### 4.6 Circulation

Panel 2, heart rate and O_2_‐pulse plotted against time, provided information about circulatory function. The aggregated panel 2 data (Figure 1b) showed that median heart rate increased during unloaded and loaded cycling and returned to baseline after exercise. Predicted maximal HR decreases with age (Karvonen et al., 1957) and should be reached during maximal exercise if the circulation is the limiting factor to exercise, which is the case in healthy individuals. In our patients, the heart rate remained well under the predicted maximal HR, indicating that there was still circulatory reserve at peak exercise.

O_2_‐pulse, that is, the volume of oxygen taken up in one heartbeat, showed a similar pattern, increasing during unloaded and loaded exercise and returning to normal afterward. O_2_‐pulse remained <80% of predicted in all our participants, except for #7, who reached this maximum level before reaching the AT. Investigating the development of O_2_‐pulse over time in each individual patient may yield information that the overall figures do not show. Three patients (#1, 2, 3) showed a decrease in O_2_‐pulse after an initial increase, which is suggestive of a decrease in SV at higher exercise levels, as can be encountered in ischemic heart disease. However, no ischemia was detected on the 3‐lead ECG of these patients, and they had no angina pectoris.

In healthy subjects, HR increases linearly with ${\dot{V}O}_{2}$ with a slope that is determined by their predicted maximums. The plot of HR against ${\dot{V}O}_{2}$ in panel 3 has the potential to identify abnormalities in this relationship, which may indicate cardiac disease. In our patients, panel 3 showed that HR increased in proportion to ${\dot{V}O}_{2}$ in most patients. In two cases (#1, 2) abnormalities in this relationship could be associated with a cause (AF and inotropic support, respectively), which was not identified by analyzing panel 2 only. We therefore, conclude that HR in panel 3 was a useful addition to identify cardiac limitations in our patient cohort.

Panel 5 of the nine‐panel plots includes noninvasive blood pressure measurements. Unfortunately, it was not possible to feed arterial blood pressure measurements from our monitors directly into the Quark respiratory gas analyzer, to be displayed in the nine‐panel plots.

### 4.7 Ventilation

In a normal diagnostic workup, panel 9 (VT against $\dot{V}E$) integrates values obtained by volumetric lung function tests with measurements during exercise. As ICU patients are often admitted acutely, lung function parameters to calculate the predicted maximum voluntary ventilation are rarely available. Without these values, ventilatory limitations are difficult to identify. Therefore, we found that in the ICU situation, panel 9 lacked much of its relevance.

$\dot{V}E$ is measured during exercise testing, and its development in time (panel 5) is indispensable to interpret the metabolic data in panel 1. $\dot{V}E$ increased during exercise in all patients (Figure 1d). In normal subjects, V_T_ increases first, and respiratory frequency later. Because $\dot{V}E$ is the product of V_T_ and respiratory frequency, plotting these components on the time axis separately might enhance insight into the sequence of events. Displaying other indices of ventilation, such as T_I_ and T_E_ over time may add further insight in ventilatory patterns.

### 4.8 Gas exchange

The strength of panel 7, displaying partial pressures of O_2_ and CO_2_ over time, is that it visually represents many of the values listed in Table 2. It is normal for P_E_O_2_ and P_ET_O_2_ to show a slight decrease during exercise (Wasserman et al., 2012). In some of our patients these values remained at the same level, which may have been due to submaximal exercise. Abnormalities in oxygenation are common in ventilated patients; at rest P_A‐a_O_2_ was elevated in five of seven patients, suggesting diffusion problems or ventilation/perfusion mismatch. During mild and moderate exercise, this difference tends to increase in healthy subjects (Hansen et al., 1967; Malmberg et al., 1987), but in our cohort, the response to exercise was inconsistent, with an increase in three and a decrease in four patients.

In normal subjects, dead space ventilation decreases during exercise (Wasserman et al., 2012) and all indices that reflect dead space ventilation (V_D_/V_T_, P_a‐ET_CO_2_ and ventilatory equivalents) would be expected to illustrate this, which indeed they did in most patients. In one patient (#5), however, elevated dead space indices at rest failed to decrease during exercise, suggesting pulmonary vascular disease. During his ICU stay, echocardiography showed a peak gradient over the tricuspid valve of 40 mmHg and a shortened pulmonary acceleration time of 86 ms, consistent with moderate pulmonary hypertension.

The ventilatory equivalents in panel 4 represent the amount of $\dot{V}E$ required for the ${\dot{V}O}_{2}$ and ${\dot{V}\text{CO}}_{2}$ at that moment. The EqCO_2_ has a mathematical relationship with V_D_/V_T_ and P_a_CO_2_, such that if the P_a_CO_2_ remains constant, EqCO_2_ is only determined by V_D_/V_T_ (Sue, 2011). In most instances below the AT, EqCO_2_ provides a reliable, noninvasive representation of dead space ventilation over time. Figure 1c shows that EqCO_2_ decreased during exercise, which is in keeping with the expected reduction in dead space ventilation.

P_ET‐E_CO_2_ is a value that reflects inhomogeneous expiration, as seen in COPD (Hansen et al., 2007). Median P_ET‐E_CO_2_ did not show a consistent change during exercise. In one patient (#7), P_ET‐E_CO_2_ was 2.3 kPa, which was above normal (Table 2). This patient was diagnosed with COPD Gold class II before admission to the ICU.

### 4.9 Proposed nine‐panel plot for patients on mechanical ventilation

During the analysis of the ergometric data, it became clear that some panels in the classical Wasserman nine‐panel montage (Wasserman et al., 2012) had limited value in patients on mechanical ventilation, while other relevant information was missing. The original panel 6 ($\dot{V}E$ vs. ${\dot{V}\text{CO}}_{2}$) provided little information, because none of the participants reached the respiratory compensation point. When used appropriately, Panel 9 (V_T_ vs. $\dot{V}E$) helps identifying ventilatory limitations, using maximal voluntary ventilation and vital capacity as references (Wasserman et al., 2012). However, without spirometric data, panel 9 lacked purpose.

In the Supporting Material, we showed several options for improvement. Some of the diagrams were based on the ERS guideline for preoperative CPET (Levett et al., 2018), intended to improve recognition of the AT. However, we were reluctant to add panels plotting the same parameters in different dimensions. Instead, we chose panels with additional ventilatory details on a time axis. The classical representation did not provide insight into subtle changes in the respiratory cycle, which may be relevant for patients who experience difficulties in separation from mechanical ventilation.

**FIGURE 2:**
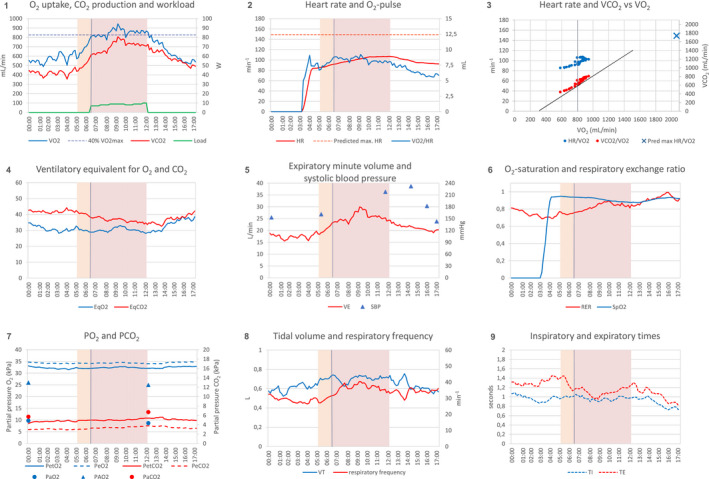
Proposed layout of nine‐panel plot for patients on mechanical ventilation. Exercise data of a mechanically ventilated patient, presented as a nine‐panel plot. The exercise test was stopped because of high systolic blood pressure. Light brown areas indicate unloaded cycling, purple areas indicate loaded cycling, vertical grey lines indicates the anaerobic threshold (at a ${\dot{V}O}_{2}$ of 800 ml/min for this patient); the tangent in panel 3 is an aid to determine the anaerobic threshold (V‐slope method). Panels 1 to 7 are identical to the nine‐panel plot proposed by Wasserman (5th Edition, 2012), with the exception of SpO_2_ (moved from panel 7 to panel 6), P_E_O_2_ and P_E_CO_2_ (added to panel 7), and P_A_O_2_ (added to panel 7). Panels 8 and 9 are new additions. Abbreviations: EqCO_2_, ventilatory equivalent for CO_2_ ($\dot{V}E$/${\dot{V}\text{CO}}_{2}$); EqO_2_, ventilatory equivalent for O_2_ ($\dot{V}E$/${\dot{V}O}_{2}$); HR, heart rate; P_a_O_2_, arterial oxygen pressure; P_a_CO_2_, arterial carbon dioxide pressure; P_A_O_2_, ideal alveolar oxygen pressure; P_E_CO_2_, mixed expired carbon dioxide pressure; P_E_O_2_, mixed expired oxygen pressure; P_ET_CO_2_, end‐tidal carbon dioxide pressure; P_ET_O_2_, end‐tidal oxygen pressure; PO_2_, partial pressure of oxygen; PCO_2_, partial pressure of carbon dioxide; RER, respiratory exchange ratio (${\dot{V}O}_{2}$/${\dot{V}\text{CO}}_{2}$); SBP, systolic blood pressure; SpO_2_, peripheral capillary oxygen saturation; T_E_, expiratory time; T_I_, inspiratory time (s); ${\dot{V}\text{CO}}_{2}$, carbon dioxide production; $\dot{V}E$, minute ventilation; ${\dot{V}O}_{2}$, oxygen uptake; VT, tidal volume

In Figure 2, the exercise data of patient #3 are used to illustrate a revised nine‐panel layout for patients on mechanical ventilation. The layout is still firmly based on Wasserman's nine‐panel layout (Wasserman et al., 2012). AT determination is still possible using both the V‐slope and the ventilatory equivalent methods (Levett et al., 2018), maintaining RER and P_ET_O_2_ as visual checks. The newly added panel 8 illustrates that V_T_ and respiratory frequency increased simultaneously during unloaded cycling, while panel 9 shows that the increase in frequency was due to decreased T_E_, while T_I_ remained constant throughout exercise.

### 4.10 Feasibility in critical illness

#### 4.10.1 Applicability

This method of exercise testing was applicable to intubated patients who were capable of active cycling and who were not immediately extubated. This subgroup (estimated to be 1:5 ventilated patients in our ICU) was of particular interest, because these patients spent a long time on ventilation (median 11 days, Table 1). In many cases, CPET provided insight into the reasons that prohibited weaning.

##### Technical

All that was required for CPET in ventilated patients, were a cycle ergometer and a metabolic analyzer. Despite this simplicity, we encountered several obstacles. First, both ergometers we used had imperfections. The ideal cycle ergometer for future research is designed for in‐bed use, with proper leg support, and suitable for passive and active cycling, like the MOTOmed. In active mode, a low start‐up load and the ability to quantify power and export ergometric data to a gas analyzer are required, as in the Lode. Second, the offline analysis was hindered by the fact that the CPET analysis tools in the Cosmed software could not be used for intubated patients. Having these tools available would simplify the nine‐panel analysis. Moreover, with these tools available at the bedside, training load could truly be guided by ergometry data, which may be superior to present practice (Whittle & San‐Millan, 2021). Third, ICU patients are routinely monitored with arterial catheters and pulse oximeters. A possibility to feed these data straight into the Omnia software would reduce the need for gadgets, and improve the analysis. Hopefully, manufacturers are willing to make their equipment more accessible for this purpose.

##### Operational

It took approximately 1 h to calibrate the equipment, go through the test phases and clean up afterward. Our safety protocol required a physiotherapist and a doctor to be present during the exercise. Operational advantages were that time for routine physiotherapy sessions was saved, and that the measured RMR provided input to estimate caloric intake. The consumption of disposables was modest and there were no other economic considerations. Due to the newness of the method, the time required for plotting the data and doing the calculations was considerable. This may be shortened by technical improvements, but diagnostic CPET involves a thorough review of different organ systems, so time must be reserved for the analysis.

##### Safety

According to recent international guidelines, “serious safety events or harms do not occur commonly during physical rehabilitation or mobilization” (Devlin et al., 2018). The safety of our exercise protocol was addressed in a previous publication (Sommers et al., 2019) and was comparable to the safety of early mobilization in general. Though active mobilization of ICU patients beyond the AT has not been described before, it is likely that in practice, many patients have crossed that boundary unknowingly. Whether this poses new safety issues, is unknown.

We encountered two adverse events, one graded as severe and one as moderate. The severe adverse event, a hypertensive response in patient #3, was transient and did not require medication. The highest systolic blood pressure was 230 mmHg, which was lower than the stop criterion of 250 mmHg suggested in the ATS guideline (American Thoracic Society and American College of Chest Physicians, 2003). However, cycling was terminated because blood pressure surpassed the more conservative stop criterion of 180 mmHg formulated in the PADIS guideline (Devlin et al., 2018). Hypertensive response to exercise is not an uncommon phenomenon in clinical exercise testing, and although we assumed that this brief period of hypertension has caused no direct harm to this patient, it is believed to be a predictor of future cardiovascular event (Kim & Ha, 2016).

The onset of atrial fibrillation (#1), graded as a moderate adverse event, may have been triggered by sympathetic stimulation during exercise. In future exercise test, we intend to add a precordial ECG lead to better identify cardiac ischemia. These two events underscore the importance of having safety criteria for physiotherapy in place (Devlin et al., 2018; Sommers et al., 2015).

### 4.11 Strengths and weaknesses

In this study, we applied a tried and tested method of CPET analysis to a new population: respiratory insufficient patients on a ventilator. The quality of the measurements was excellent with minimal burden to the patient, owing to the fact that endotracheal tubes provided gas sampling without leakage, and arterial catheters were already in place. As part of the ICU practice, other vital signs, not used for the exercise test, were monitored strictly, providing a high level of participant safety.

In most instances, the available reference values came from measurements of subjects in upright positions and breathing through face masks, while our patients were supine and intubated, which was an inherent weakness of this study. Two other weaknesses that we could not address were: (1) dead space in supine position is less than in sitting position, at least in rest (Riley et al., 1959), and (2) the instrumental dead space in intubated subjects is smaller than the volume of dead space that is bypassed by the intubation (Chadda et al., 2002). Both effects may have caused an underestimation of the V_D_/V_T_ and ventilatory equivalents in our cohort.

Another weakness was that our patient cohort was small and heterogeneous. However, the purpose of this project was not to provide a diagnostic standard for critically ill patients; much larger studies will be required for that. The main purpose of the project was to investigate whether it could be done at all, and what insights it might yield. Whether critical care ergometry has diagnostic value in explaining why certain patients are difficult to wean from mechanical ventilation remains to be investigated.

## 5 CONCLUSION

By analyzing nine‐panel plots of ventilated patients recovering from critical illness, following rules laid out in diagnostic CPET, we provided a proof of concept of critical care ergometry. Even though ${\dot{V}O}_{2\max}$ was not achieved, many relevant parameters and patterns were derived from the test results and it was possible to identify the limiting factors to exercise in a majority of cases. Though the exercise protocol and the technical equipment can still be improved, our experience was that CPET in patients on mechanical ventilation was feasible.

## ETHICS STATEMENT

The study protocol was submitted to the Medical Ethics Review Committee as an observational study during physiotherapy treatment in which increasing bed‐based cycling was provided and cardiorespiratory parameters were measured. The Ethical Review Board of Isala Clinics had no objections to the study protocol.

## CONFLICT OF INTEREST

None of the authors has any conflicting interests.

## AUTHOR CONTRIBUTION

HvdO, EK, and SZ contributed to the conception and design of this study; HvdO, MK, AO, EK, SZ, and ER developed and performed the clinical exercise tests; HvdO, MK, and BL prepared and interpreted the nine‐panel plots; the first draft of the manuscript was written by HvdO and MK; all authors read and approved the final version and agree to be accountable for all aspects of this work.

## Supporting information



Supplementary Material

## References

[phy215213-bib-0001] Ainsworth, B. E. , Haskell, W. L. , Herrmann, S. D. , Meckes, N. , Bassett, D. R. , Tudor‐Locke, C. , Greer, J. L. , Vezina, J. , Whitt‐Glover, M. C. , & Leon, A. S. (2011). Compendium of physical activities: A second update of codes and MET values. Medicine and Science in Sports and Exercise, 43, 1575–1581. 10.1249/MSS.0b013e31821ece12 21681120

[phy215213-bib-0002] Akoumianaki, E. , Dousse, N. , Lyazidi, A. , Lefebvre, J. C. , Graf, S. , Cordioli, R. L. , Rey, N. , Richard, J. M. , & Brochard, L. (2017). Can proportional ventilation modes facilitate exercise in critically ill patients? A physiological cross‐over study: Pressure support versus proportional ventilation during lower limb exercise in ventilated critically ill patients. Annals of Intensive Care, 7, 64. 10.1186/s13613-017-0289-y 28608135 PMC5468357

[phy215213-bib-0003] American Thoracic Society and American College of Chest Physicians . (2003). ATS/ACCP statement on cardiopulmonary exercise testing. American Journal of Respiratory and Critical Care Medicine. 167, 211–277. 10.1164/rccm.167.2.211 12524257

[phy215213-bib-0004] Carnes, A. J. , & Mahoney, S. E. (2018). Polarized vs. high intensity multimodal training in recreational runners. International Journal of Sports Physiology and Performance, 1–28. 10.1123/ijspp.2018-0040 29952662

[phy215213-bib-0005] Chadda, K. , Louis, B. , Benaissa, L. , Annane, D. , Gajdos, P. , Raphael, J. C. , & Lofaso, F. (2002). Physiological effects of decannulation in tracheostomized patients. Intensive Care Medicine, 28, 1761–1767. 10.1007/s00134-002-1545-6 12447520

[phy215213-bib-0006] Devlin, J. W. , Skrobik, Y. , Gelinas, C. , Needham, D. M. , Slooter, A. J. C. , Pandharipande, P. P. , Watson, P. L. , Weinhouse, G. L. , Nunnally, M. E. , Rochwerg, B. , Balas, M. C. , van den Boogaard, M. , Bosma, K. J. , Brummel, N. E. , Chanques, G. , Denehy, L. , Drouot, X. , Fraser, G. L. , Harris, J. E. , … Alhazzani, W. (2018). Clinical practice guidelines for the prevention and management of pain, agitation/sedation, delirium, immobility, and sleep disruption in adult patients in the ICU. Critical Care Medicine, 46, e825–e873. 10.1097/CCM.0000000000003299 30113379

[phy215213-bib-0007] Doiron, K. A. , Hoffmann, T. C. , & Beller, E. M. (2018). Early intervention (mobilization or active exercise) for critically ill adults in the intensive care unit. Cochrane Database Systematic Review, 3, CD010754. 10.1002/14651858.CD010754.pub2 PMC6494211 29582429

[phy215213-bib-0008] European Respiratory Society . (1997). Anonymous clinical exercise testing with reference to lung diseases: Indications, standardization and interpretation strategies. ERS task force on standardization of clinical exercise testing. European Respiratory Society. European Respiratory Journal, 10, 2662–2689. 10.1183/09031936.97.10112662 9426113

[phy215213-bib-0009] Faisy, C. , Guerot, E. , Diehl, J. L. , Labrousse, J. , & Fagon, J. Y. (2003). Assessment of resting energy expenditure in mechanically ventilated patients. American Journal of Clinical Nutrition, 78, 241–249. 10.1093/ajcn/78.2.241 12885704

[phy215213-bib-0010] Hansen, J. E. , Casaburi, R. , Cooper, D. M. , & Wasserman, K. (1988). Oxygen uptake as related to work rate increment during cycle ergometer exercise. European Journal of Applied Physiology and Occupational Physiology, 57, 140–145. 10.1007/BF00640653 3349978

[phy215213-bib-0011] Hansen, J. E. , Sue, D. Y. , & Wasserman, K. (1984). Predicted values for clinical exercise testing. American Review of Respiratory Disease, 129, 49. 10.1164/arrd.1984.129.2P2.S49 6421218

[phy215213-bib-0012] Hansen, J. E. , Ulubay, G. , Chow, B. F. , Sun, X. G. , & Wasserman, K. (2007). Mixed‐expired and end‐tidal CO_2_ distinguish between ventilation and perfusion defects during exercise testing in patients with lung and heart diseases. Chest, 132(3), 977–983. 10.1378/chest.07-0619 17573506

[phy215213-bib-0013] Hansen, J. E. , Vogel, J. A. , Stelter, G. P. , & Consolazio, C. F. (1967). Oxygen uptake in man during exhaustive work at sea level and high altitude. Journal of Applied Physiology, 23, 511–522. 10.1152/jappl.1967.23.4.511 6053677

[phy215213-bib-0014] Heydenreich, J. , Schutz, Y. , Melzer, K. , & Kayser, B. (2019). Comparison of conventional and individualized 1‐MET values for expressing maximum aerobic metabolic rate and habitual activity related energy expenditure. Nutrients, 11(2), 458. 10.3390/nu11020458 PMC6412759 30813275

[phy215213-bib-0015] Heyland, D. K. , Stapleton, R. D. , Mourtzakis, M. , Hough, C. L. , Morris, P. , Deutz, N. E. , Colantuoni, E. , Day, A. , Prado, C. M. , & Needham, D. M. (2016). Combining nutrition and exercise to optimize survival and recovery from critical illness: Conceptual and methodological issues. Clinical Nutrition, 35, 1196–1206. 10.1016/j.clnu.2015.07.003 26212171

[phy215213-bib-0016] Karvonen, M. J. , Kentala, E. , & Mustala, O. (1957). The effects of training on heart rate: A longitudinal study. Annales Medicinae Experimentalis et Biologiae Fenniae, 35, 307–315. 13470504

[phy215213-bib-0017] Kim, D. , & Ha, J.‐W. (2016). Hypertensive response to exercise: Mechanisms and clinical implication. Clinical Hypertension, 22(1). 10.1186/s40885-016-0052-y PMC4962449 27468357

[phy215213-bib-0018] Klainman, E. , Yosey, C. , Caspi, A. , Landau, A. , Vishnitzer, R. , & Fink, G. (2008). Recovery kinetics of oxygen uptake in patients with various degrees of coronary artery disease. Journal of Clinical and Basic Cardiology, 10, 16–19.

[phy215213-bib-0019] Kwan, M. , Woo, J. , & Kwok, T. (2004). The standard oxygen consumption value equivalent to one metabolic equivalent (3.5 ml/min/kg) is not appropriate for elderly people. International Journal of Food Sciences and Nutrition, 55, 179–182. 10.1080/09637480410001725201 15223593

[phy215213-bib-0020] Levett, D. Z. H. , Jack, S. , Swart, M. , Carlisle, J. , Wilson, J. , Snowden, C. , Riley, M. , Danjoux, G. , Ward, S. A. , Older, P. , & Grocott, M. P. W. ; Perioperative Exercise Testing and Training Society, (POETTS) . (2018). Perioperative cardiopulmonary exercise testing (CPET): Consensus clinical guidelines on indications, organization, conduct, and physiological interpretation. British Journal of Anaesthesia, 120, 484–500. 29452805 10.1016/j.bja.2017.10.020

[phy215213-bib-0021] Malmberg, P. , Hedenstrom, H. , & Fridriksson, H. V. (1987). Reference values for gas exchange during exercise in healthy nonsmoking and smoking men. Bulletin Européen de Physiopathologie Respiratoire, 23, 131–138. 3111570

[phy215213-bib-0022] National Cancer Institute . (2020). Common Terminology Criteria for Adverse Events (CTCAE) v5.0 [Online].

[phy215213-bib-0023] Radtke, T. , Crook, S. , Kaltsakas, G. , Louvaris, Z. , Berton, D. , Urquhart, D. S. , Kampouras, A. , Rabinovich, R. A. , Verges, S. , Kontopidis, D. , Boyd, J. , Tonia, T. , Langer, D. , De Brandt, J. , Goertz, Y. M. J. , Burtin, C. , Spruit, M. A. , Braeken, D. C. W. , Dacha, S. , … Hebestreit, H. (2019). ERS statement on standardisation of cardiopulmonary exercise testing in chronic lung diseases. European Respiratory Reviews, 28, 1–25. 10.1183/16000617.0101-2018 PMC9488712 31852745

[phy215213-bib-0024] Riley, R. L. , Permutt, S. , Said, S. , Godfrey, M. , Cheng, T. O. , Howell, J. B. , & Shepard, R. H. (1959). Effect of posture on pulmonary dead space in man. Journal of Applied Physiology, 14, 339–344. 10.1152/jappl.1959.14.3.339 13654159

[phy215213-bib-0025] Roca, J. , Whipp, B. J. , Agustí, A. G. N. , Anderson, S. D. , Casaburi, R. , Cotes, J. E. , Donner, C. F. , Estenne, M. , Folgering, H. , Higenbottam, T. W. , Killian, K. J. , Palange, P. , Patessio, A. , Prefaut, C. , Sergysels, R. , Wagner, P. D. , & Weisman, I. (1997). Clinical exercise testing with reference to lung diseases: Indications, standardization and interpretation strategies. European Respiratory Journal, 10, 2662–2689. 10.1183/09031936.97.10112662 9426113

[phy215213-bib-0026] Skjorten, I. , Ankerstjerne, O. A. W. , Trebinjac, D. , Bronstad, E. , Rasch‐Halvorsen, O. , Einvik, G. , Lerum, T. V. , Stavem, K. , Anne, E. , & Ingul, C. B. (2021). Cardiopulmonary exercise capacity and limitations 3 months after COVID‐19 hospitalisation. European Respiratory Journal, 2100996. 10.1183/13993003.00996-2021 PMC8247555 34210791

[phy215213-bib-0027] Sommers, J. , Engelbert, R. H. , Dettling‐Ihnenfeldt, D. , Gosselink, R. , Spronk, P. E. , Nollet, F. , & van der Schaaf, M. (2015). Physiotherapy in the intensive care unit: an evidence‐based, expert driven, practical statement and rehabilitation recommendations. Clinical Rehabilitation, 29, 1051–1063. 10.1177/0269215514567156 25681407 PMC4607892

[phy215213-bib-0028] Sommers, J. , Klooster, E. , Zoethout, S. B. , van den Oever, H. L. A. , Nollet, F. , Tepaske, R. , Horn, J. , Engelbert, R. H. H. , & van der Schaaf, M. (2019). Feasibility of exercise testing in patients who are critically ill: A prospective, observational multicenter study. Archives of Physical Medicine and Rehabilitation, 100, 239–246. 30142315 10.1016/j.apmr.2018.07.430

[phy215213-bib-0029] Stelken, A. M. , Younis, L. T. , Jennison, S. H. , Miller, D. D. , Miller, L. W. , Shaw, L. J. , Kargl, D. , & Chaitman, B. R. (1996). Prognostic value of cardiopulmonary exercise testing using percent achieved of predicted peak oxygen uptake for patients with ischemic and dilated cardiomyopathy. Journal of the American College of Cardiology, 27, 345–352. 10.1016/0735-1097(95)00464-5 8557904

[phy215213-bib-0030] Sue, D. Y. (2011). Excess ventilation during exercise and prognosis in chronic heart failure. American Journal of Respiratory and Critical Care Medicine, 183, 1302–1310. 10.1164/rccm.201006-0965CI 21257789

[phy215213-bib-0031] Wasserman, K. , Hansen, J. E. , Sue, D. Y. , & Stringer, W. (2012). Principles of exercise testing and interpretation: including pathophysiology and clinical applications (p. 572). Wolters Kluwer Health/Lippincott Williams & Wilkins.

[phy215213-bib-0032] Wasserman, K. , Whipp, B. J. , & Castagna, J. (1974). Cardiodynamic hyperpnea: hyperpnea secondary to cardiac output increase. Journal of Applied Physiology, 36, 457–464. 10.1152/jappl.1974.36.4.457 4820330

[phy215213-bib-0033] Whipp, B. J. , & Wasserman, K. (1969). Efficiency of muscular work. Journal of Applied Physiology, 26, 644–648. 10.1152/jappl.1969.26.5.644 5781619

[phy215213-bib-0034] Whittle, J. , & San‐Millan, I. (2021). Objective assessment of metabolism and guidance of ICU rehabilitation with cardiopulmonary exercise testing. Current Opinion in Critical Care, 27, 390–398. 10.1097/MCC.0000000000000843 33973897

[phy215213-bib-0035] Wischmeyer, P. E. , Puthucheary, Z. , San Millan, I. , Butz, D. , & Grocott, M. P. W. (2017). Muscle mass and physical recovery in ICU: innovations for targeting of nutrition and exercise. Current Opinion in Critical Care, 23, 269–278. 10.1097/MCC.0000000000000431 28661414 PMC5599154

