# Supplementary material for: Feasibility of critical care ergometry: Exercise data of patients on mechanical ventilation analyzed as nine‐panel plots
Source: Physiol Rep. 2022 Mar 13;10(5):e15213. doi: 10.14814/phy2.15213 (PMC8918698; doi:10.14814/phy2.15213)
Supplement: Supplementary file 1 — Supplementary Material [file PHY2-10-e15213-s001.pdf]

Feasibility of critical care ergometry: exercise data of patients on mechanical ventilation analyzed as nine-panel plots

Physiological Reports

Huub L.A. van den Oever, Mert Kök, Aloys Oosterwegel, Emily Klooster,

Siebrand Zoethout, Erwin Ruessink, Bas Langeveld

Corresponding author: H.L.A. van den Oever, Intensive Care Unit,

Deventer Hospital, Deventer, Netherlands. email: h.vandenoever@dz.nl

Supporting material for patient #1 to #7, containing:

Nine-panel plots, additional plots and full ergometry reports

Contents:

Patient #1

Nine-panel plots 3
Additional plots 4
Full ergometry report 5

Patient #2

Nine-panel plots 6
Additional plots 7
Full ergometry report 8

Patient #3

Nine-panel plots 9
Additional plots 10
Full ergometry report 11

Patient #4

Nine-panel plots 12
Additional plots 13
Full ergometry report 14

Patient #5

Nine-panel plots 15
Additional plots 16
Full ergometry report 17

Patient #6

Nine-panel plots 18
Additional plots 19
Full ergometry report 20

Patient #7

Nine-panel plots 21
Additional plots 22
Full ergometry report 23

Patient #1
Wasserman 9-panel plots

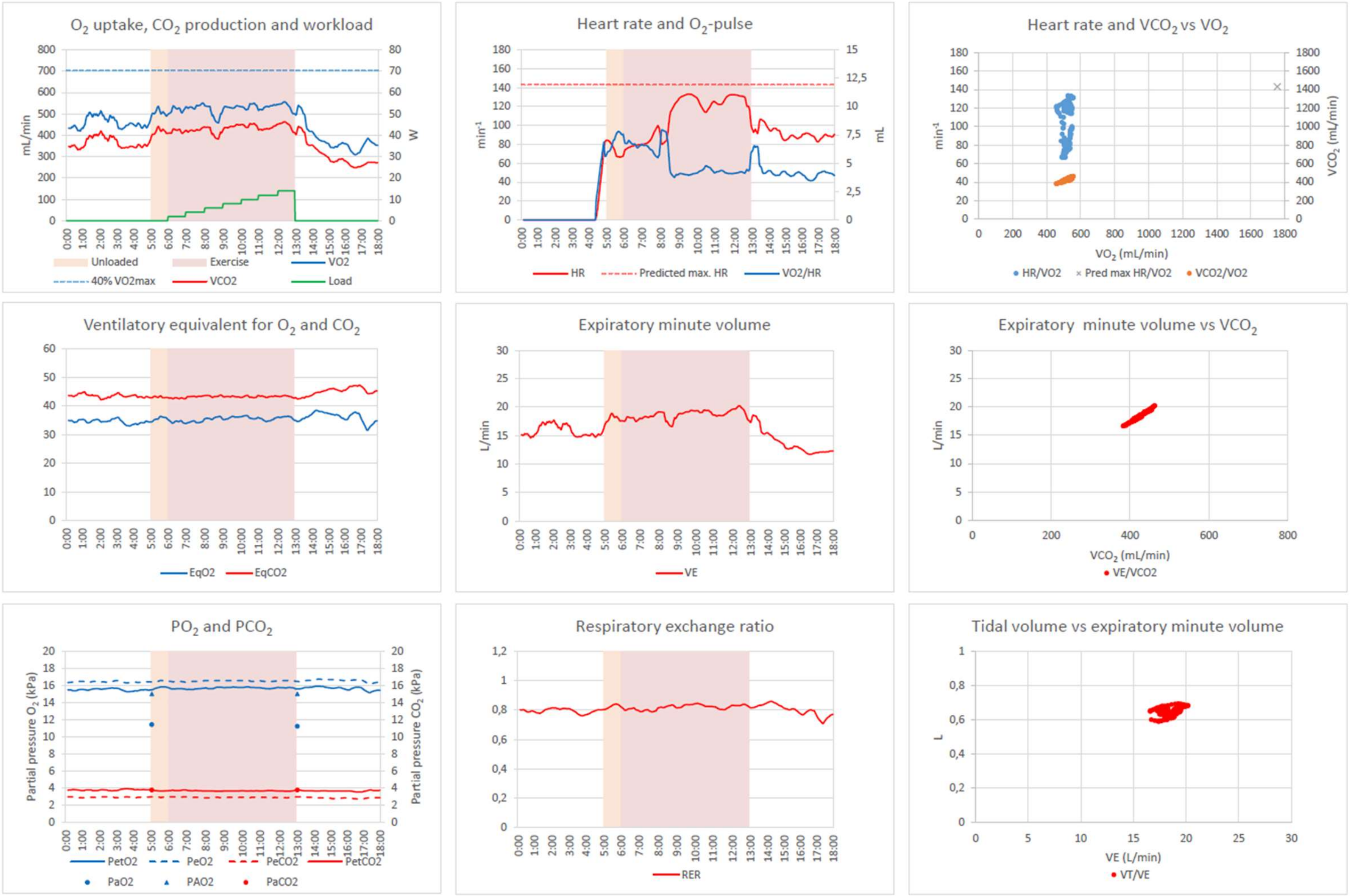

Patient #1

Additional plots ventilation

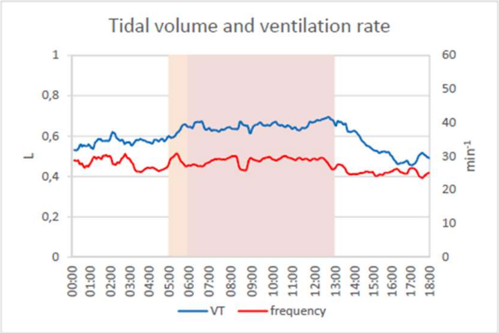

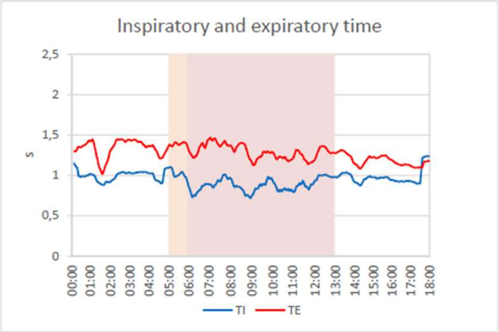

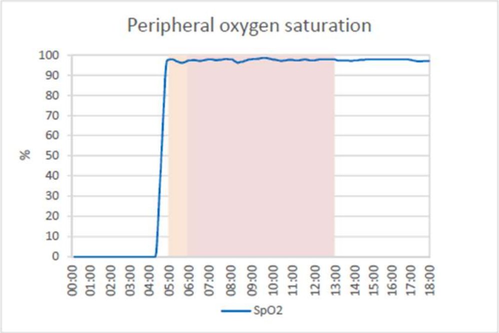

Additional plots anaerobic threshold

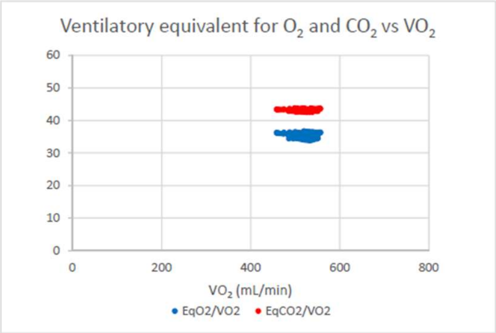

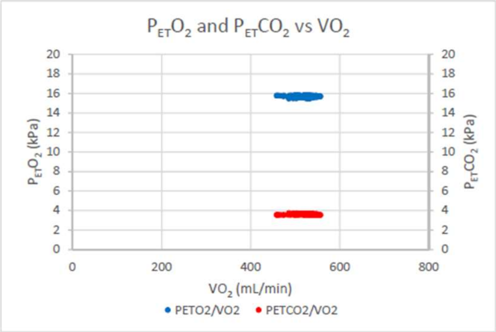

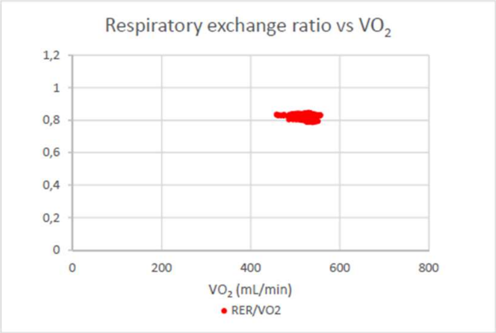

Patient 1

Ergometer: Lode
Age: 77
Sex: male
Height (cm): 179
Weight (kg): 78
BMI (kg/m²): 24.3
Relevant comorbidity: Moderate ARDS
Heart rhythm: DDDR with underlying paroxysmal atrial fibrillation
Relevant medication: metoprolol

Admission diagnosis: Pneumosepsis
Admission day (days): 18
Intubation day (days): 17
Airway: Shiley percutaneous tracheostomy tube 8.0
Ventilation mode: Pressure support ventilation
Pressure support (cm H_2O): 8
PEEP (cm H_2O): 6
F_iO_2 (%): 21

Work and metabolism

- Predicted $\dot{V}O_{2max}$: 1757 mL/min
- $\dot{V}O_2$ at rest: 459 mL/min – 5.88 mL/kg/min (high) – 26.1% of predicted $\dot{V}O_{2max}$
- Maximal workload (W): 14
- $\dot{V}O_{2peak}$: 557 mL/min – 7.14 mL/kg/min (low) – 31.7% of predicted $\dot{V}O_{2max}$
- MET: 1.21
- O_2 consumption increased rapidly during unloaded exercise and reached a normal level
- Increase in O_2 consumption in proportion to workload during exercise was low
- Anaerobic threshold was not reached graphically
- RER at $\dot{V}O_{2peak}$: 0.83 (low)
- Lactate changed from 1.5 mmol/L (within normal range) before exercise to 1.6 mmol/L (within normal range) directly after exercise

Circulation

- HR at rest: 80 bpm
- During exercise HR gradually increased, then showed a sudden decrease, before going into atrium fibrillation
- Predicted maximum HR: 143 bpm
- Maximum HR: 133 bpm
- HR reserve: 10 bpm
- Maximum HR correlated with $\dot{V}O_{2peak}$
- Predicted maximum O_2-pulse: 12.3 mL/heartbeat
- O_2-pulse increased during unloaded cycling, then decreased when atrial fibrillation set in to 4.25 mL/heartbeat at peak exercise (very low) – 34.6% of predicted O_2-pulse
- Blood pressure had a normal course during exercise
- ECG changed from a paced rhythm to atrial fibrillation during exercise

Ventilation

- Maximal ventilation: 20.2 L/min (at $\dot{V}O_{2peak}$: 20.2 L/min)
- Maximal voluntary volume: unknown
- Breathing reserve: unknown
- Maximal tidal volume: 0.69 L (at $\dot{V}O_{2peak}$: 0.68 L)

Gas exchange

- P_aO_2 at rest was 11.4 kPa (within normal range) and had a similar value of 11.2 kPa directly after exercise
- P_aCO_2 at rest was 3.7 kPa (below normal range) and had a similar value of 3.7 kPa directly after exercise
- Alveolar-arterial PO_2 gradient at rest was 3.88 kPa and had a higher value of 4.21 kPa (within normal range) directly after exercise
- $P_{E(T)}O_2$ and $P_{E(T)}CO_2$ remained unchanged during exercise
- V_D/V_T at rest was 0.22 (within normal range) and 0.24 directly after exercise (within normal range)
- $EqCO_2$ at $\dot{V}O_{2peak}$ was 43.6 (above normal range)
- $P_{(a-ET)}CO_2$ at rest was -0.03 kPa (within normal range) and had a slightly higher value of 0.01 kPa directly after exercise (within normal range)
- $P_{(ET-E)}CO_2$ at rest was 0.86 kPa and had a lower value of 0.75 kPa directly after exercise (within normal range)

Conclusion:

Basal metabolism was elevated (which is common after sepsis). Oxygen consumption increased normally during unloaded cycling, but failed to increase further, despite a reasonable amount of external work. Exercise tolerance was reduced to 31.7% of predicted $\dot{V}O_2$max, and the anaerobic threshold was not reached.
Oxygen pulse increased normally during unloaded cycling, but decreased sharply when atrial fibrillation developed during loaded exercise, suggesting poor stroke volume during AF and a cardiac limitation to further workload.
Gas exchange values were essentially normal at rest (which is unexpected after ARDS), except for a high ventilatory equivalent of CO_2, which might be due to hyperventilation and a resulting low arterial PCO_2. During exercise, gas exchange did not improve (which it normally does due to improved V/P matching).
In this patient the exercise tolerance was reduced, and the results suggested a circulatory limitation.

Patient #2
Wasserman 9-panel plot

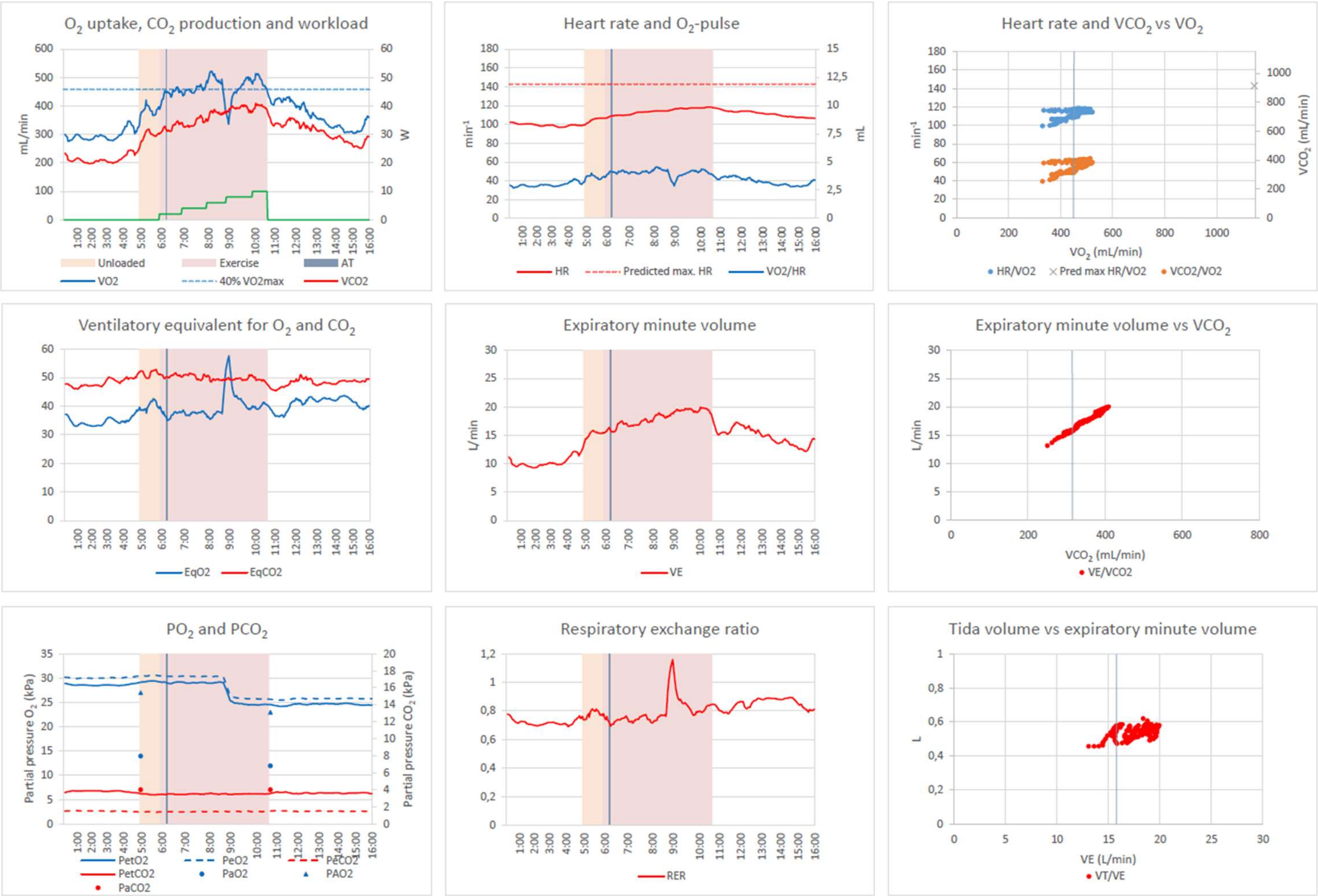

Patient #2

Additional plots ventilation

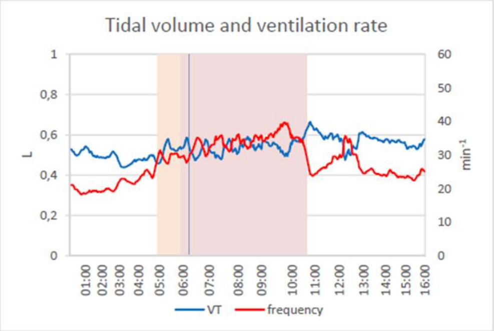

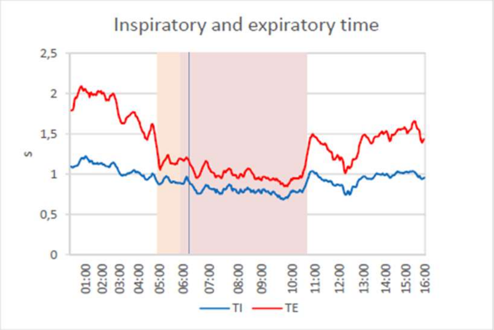

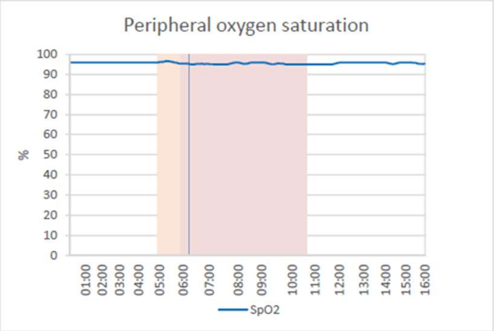

Additional plots anaerobic threshold

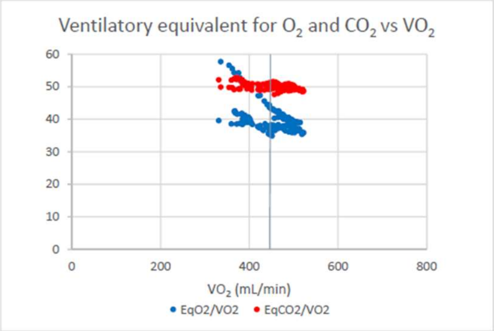

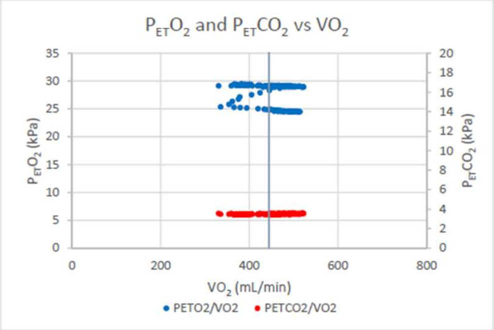

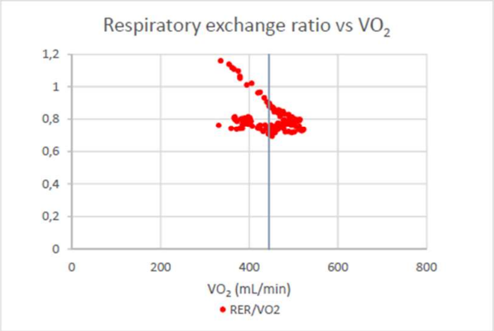

Patient 2

Ergometer: Lode
Age: 77
Sex: female
Height (cm): 164
Weight (kg): 82
BMI (kg/m^2): 30.5
Relevant comorbidity: Moderate ARDS
Heart rhythm: sinus rhythm
Relevant medication: norepinephrine and milrinone

Admission diagnosis: Pneumosepsis
Admission day (days): 2
Intubation day (days): 2
Airway: Endotracheal tube (size 8)
Ventilation mode: pressure support ventilation
Pressure support (cm H_2O): 6
PEEP (cm H_2O): 8
F_iO_2 (%): 35

Work and metabolism
- Predicted $\dot{V}O_{2max}$: 1145 mL/min
- $\dot{V}O_2$ at rest: 297 mL/min – 3.62 mL/kg/min (high) – 25.9% of predicted $\dot{V}O_{2max}$
- Maximal workload (W): 10
- $\dot{V}O_{2peak}$: 514 mL/min – 6.27 mL/kg/min (low) – 44.9% of predicted $\dot{V}O_{2max}$
- MET: 1.73
- O_2 consumption increased rapidly during unloaded exercise. A normal plateau was not reached
- Increase in O_2 consumption in proportion to workload during exercise was initially normal, but leveled off towards the end of exercise
- V-slope method and a gradual increase in RER suggest that the anaerobic threshold was reached at the end of unloaded cycling, at a $\dot{V}O_2$ of 450 L/min
- RER at $\dot{V}O_{2peak}$: 0.80 (low)
- Lactate changed from 2.4 mmol/L (above normal range) before exercise to 3.1 mmol/L directly after exercise

Circulation
- HR at rest: 99 bpm
- Predicted maximum HR: 143 bpm
- Maximum HR: 118 bpm
- HRR: 25 bpm
- HR/ $\dot{V}O_2$-slope was steep; possibly related to inotropic support
- Predicted maximum O_2-pulse: 8.0 mL/heartbeat
- O_2-pulse: slight decrease towards the end of exercise, 4.38 mL/heartbeat at peak exercise (low) – 54.7% of predicted O_2-pulse
- Blood pressure had a normal course during exercise
- ECG remained unchanged

Ventilation
- Maximal ventilation: 20.0 L/min (at $\dot{V}O_{2peak}$: 20.0 L/min)
- Maximal voluntary volume: unknown
- Breathing reserve: unknown
- Maximal tidal volume: 0.62 L (at $\dot{V}O_{2peak}$: 0.58 L)

Gas exchange
- After approximately three minutes of loaded exercise, the F_iO_2 setting on the ventilator was reduced from 35% to 30% causing an artefact in the measurement of $\dot{V}O_2$, but not $\dot{V}CO_2$
- P_aO_2 at rest was 14.6 kPa (above normal range) and had a lower value of 12.2 kPa directly after exercise (within normal range)
- P_aCO_2 at rest was 4.7 kPa (within normal range) and had a similar value of 4.5 kPa directly after exercise
- Alveolar-arterial PO_2 gradient at rest was 12.7 kPa and had a lower value of 11.1 kPa directly after exercise (above normal range; even with correction for an altered/lower F_iO_2)
- $P_{E(T)}O_2$ abruptly decreased during exercise due to a reduction of F_iO_2. $P_{E(T)}CO_2$ remained unchanged during exercise.
- V_D/V_T at rest was 0.44 (above normal range) and 0.42 directly after exercise (above normal range)
- $EqCO_2$ at $\dot{V}O_{2peak}$ was 48.9 (above normal range)
- $P_{(a-ET)}CO_2$ at rest was 0.89 kPa (above normal range) and had a slightly higher value of 0.97 kPa directly after exercise (above normal range)
- $P_{(ET-E)}CO_2$ at rest was 1.17 kPa and had a lower value of 0.92 kPa directly after exercise (within normal range)

Conclusion:

Basal metabolism was elevated (which is common after sepsis). Oxygen consumption increased normally during unloaded and loaded cycling, reaching a peak oxygen consumption of 44.9% of predicted, which was relatively high for a critically ill patient. The relation between work and O_2 consumption appeared normal. At an oxygen consumption of 450 ml/min the anaerobic threshold was reached, which was confirmed by an increase in serum lactate at the end of exercise.
Heart rate increased normally in proportion to oxygen consumption, but at the end of exercise the oxygen pulse appeared to decrease, which might indicate a reduction in stroke volume. Though blood pressure and ECG did not show any signs of cardiac ischemia, there appeared to be a cardiac limitation to exercise at a heart rate of 120/min.
The A-a gradient of oxygen was very high, suggesting a diffusion problem or significant shunting of the pulmonary circulation. An elevated dead space fraction supported poor ventilation perfusion matching. During exercise, dead space decreased slightly (which is a normal response). Although this response was not accompanied by a reduction in arterial-endtidal PCO_2 difference, it was supported by a gradual reduction in the ventilatory equivalent of CO_2.
In summary, exercise tolerance was reduced, the result suggested a circulatory limitation and impaired gas exchange.

Patient #3
Wasserman 9-panel plots

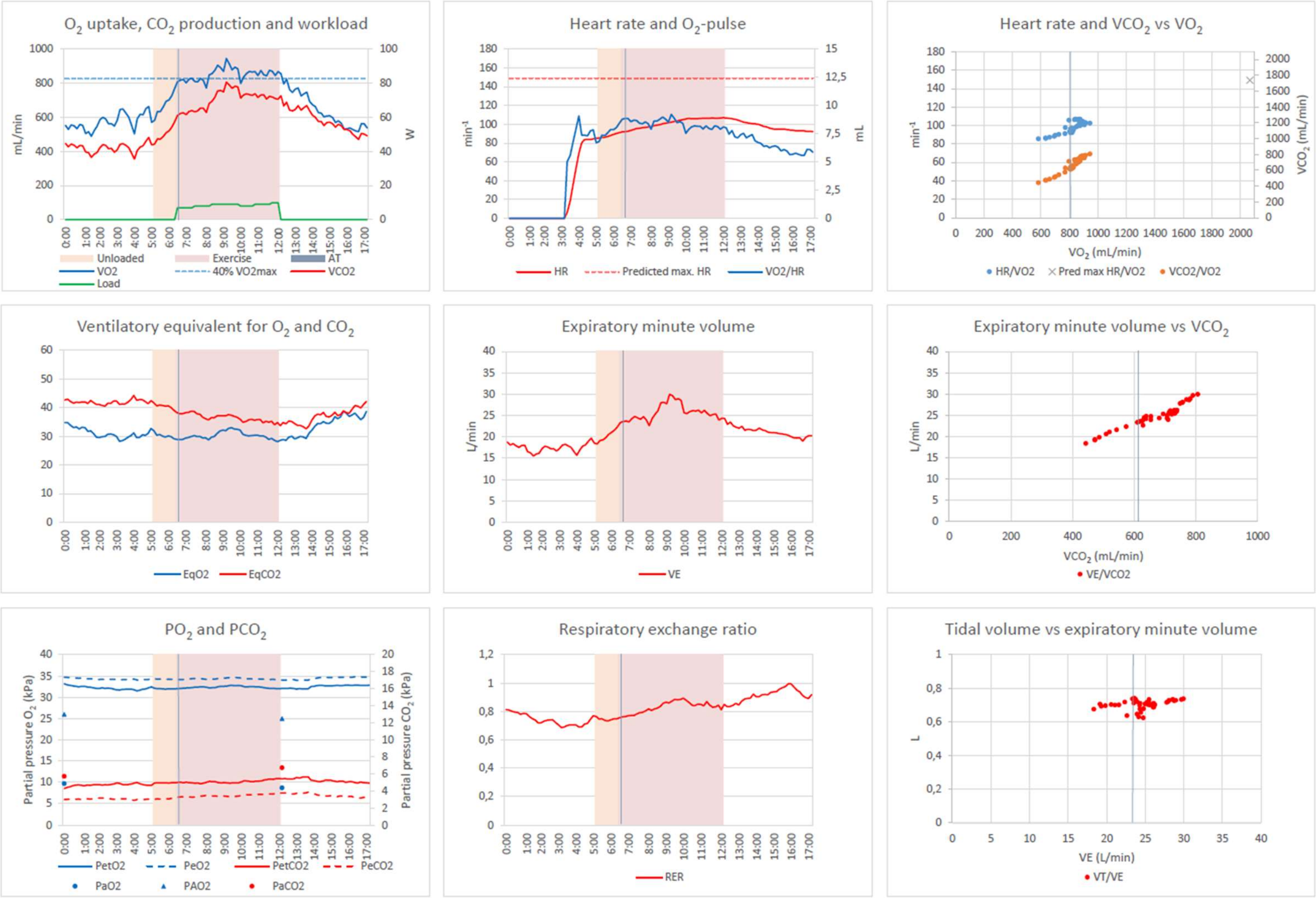

Patient #3
Additional plots ventilation

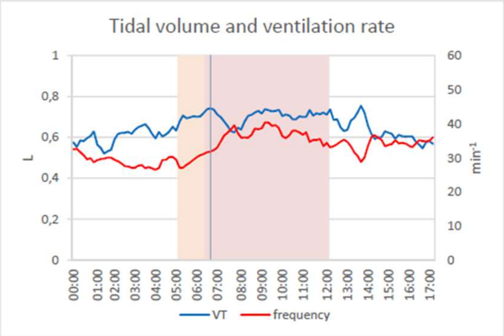

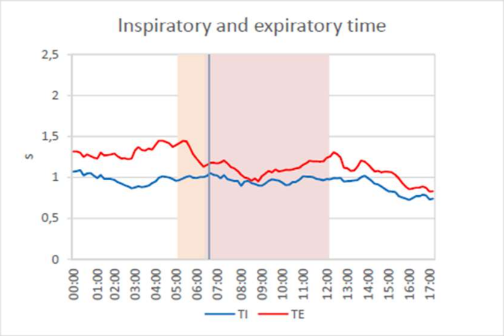

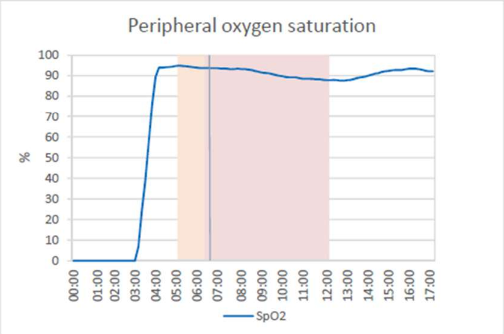

Additional plots anaerobic threshold

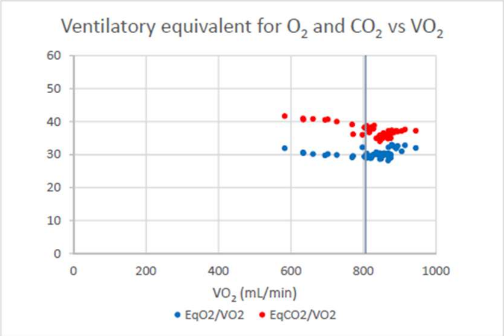

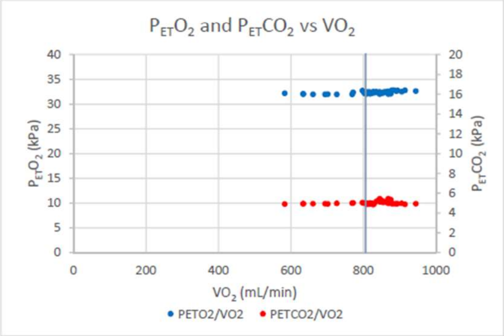

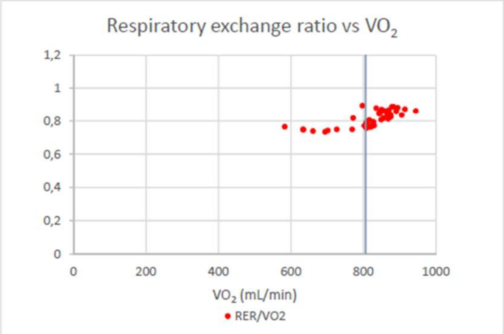

Patient 3

Ergometer: MOTOmed
Age: 71
Sex: male
Height (cm): 183
Weight (kg): 85
BMI (kg/m^2): 25.4
Relevant comorbidity: none
Heart rhythm: sinus rhythm
Relevant medication: none

Admission diagnosis: Pneumosepsis
Admission day (days): 6
Intubation day (days): 4
Airway: tube (size 8)
Ventilation mode: pressure support ventilation
Pressure support (cm H_2O): 8
PEEP (cm H_2O): 8
F_iO_2 (%): 35

Work and metabolism
- Predicted $\dot{V}O_{2max}$: 2067 mL/min
- $\dot{V}O_2$ at rest: 572 mL/min – 6.73 mL/kg/min (high) – 27.7% of predicted $\dot{V}O_{2max}$
- Maximal workload (W): 10
- $\dot{V}O_{2peak}$: 945 mL/min – 11.1 mL/kg/min (low) – 45.7% of predicted $\dot{V}O_{2max}$
- MET: 1.65
- O_2 consumption increased rapidly during unloaded exercise
- Increase in O_2 consumption in proportion to workload during exercise was normal
- The V-slope method and an increase in RER suggest that the anaerobic threshold was reached at a $\dot{V}O_2$ of 800 mL/min, corresponding to 38.7% of predicted $\dot{V}O_{2max}$
- RER at $\dot{V}O_{2peak}$: 0.86 (low)
- Lactate changed from 0.9 mmol/L (within normal range) before exercise to 3.1 mmol/L at directly after exercise (above normal range)

Circulation
- HR at rest: 84 bpm
- Predicted maximum HR: 149 bpm
- Maximum HR: 107 bpm
- HRR: 42 bpm
- Maximum HR did not correlate with $\dot{V}O_{2peak}$
- Predicted maximum O_2-pulse: 13.9 mL/heartbeat
- O_2-pulse decreased towards the end of exercise, 9.22 mL/heartbeat at peak exercise (low) – 66.4% of predicted O_2-pulse
- Hypertensive response during exercise, 230 mmHg systolic
- ECG remained unchanged

Ventilation
- Maximal ventilation: 29.9 L/min (at $\dot{V}O_{2peak}$: 29.9 L/min)
- Maximal voluntary volume: unknown
- Breathing reserve: unknown
- Maximal tidal volume: 0.74 L (at $\dot{V}O_{2peak}$: 0.74 L)

Gas exchange
- P_aO_2 at rest was 9.7 kPa (within normal range) and had a lower value of 8.7 kPa directly after exercise (below normal range)
- P_aCO_2 at rest was 5.7 kPa (within normal range) and had a higher value of 6.7 kPa directly after exercise (above normal range)
- Alveolar-arterial PO_2 gradient at rest was 16.4 kPa and had a similar value of 16.7 kPa directly after exercise (above normal range)
- $P_{E(T)}O_2$ remained unchanged during exercise, whilst $P_{E(T)}CO_2$ increased
- V_D/V_T at rest was 0.47 (above normal range) and 0.44 directly after exercise (above normal range)
- $EqCO_2$ at $\dot{V}O_{2peak}$ was 37.1 (above normal range)
- $P_{(a-ET)}CO_2$ at rest was 1.01 kPa (above normal range) and had a slightly higher value of 1.27 kPa directly after exercise (above normal range)
- $P_{(ET-E)}CO_2$ at rest was 1.65 kPa and had a similar value of 1.67 kPa directly after exercise (within normal range)

Conclusion:

Basal metabolism was elevated (which is common after sepsis). Oxygen consumption increased normally during unloaded and loaded cycling, reaching a peak oxygen consumption of 45.7% of predicted (which was relatively good for a critically ill patient). The relation between work and O_2 consumption appeared normal. At an oxygen consumption of 800 ml/min (at 38.7% of predicted $\dot{V}O_2$max) the anaerobic threshold was reached, which was confirmed by an increase in serum lactate after exercise.
A remarkable observation was that at the end of exercise, the PO_2 had decreased and the PCO_2 had increased, without an accompanying further increase in minute volume. Due to this unexpected rise in arterial PCO_2, the ventilatory equivalent for CO_2 did not increase after the anaerobic threshold.
Heart rate increased normally in proportion to oxygen consumption, but above a heart rate of 100/min, the oxygen pulse appeared to decrease, which might indicate a reduction in stroke volume. This might indicate some degree of diastolic dysfunction, as blood pressure was high and ECG did not show any signs of cardiac ischemia.
The A-a gradient of oxygen was very high, suggesting a diffusion problem or significant shunting of the pulmonary circulation. An elevated dead space fraction supported poor ventilation perfusion matching. During exercise, dead space decreased slightly (which is a normal response). Although this response was not accompanied by a reduction in arterial-endtidal PCO_2 difference, it was supported by a gradual reduction in the ventilatory equivalent of CO_2.
In summary, exercise tolerance was reduced. The results suggested both circulatory and ventilatory limitations, and impaired gas exchange.

Patient #4
Wasserman 9-panel plots

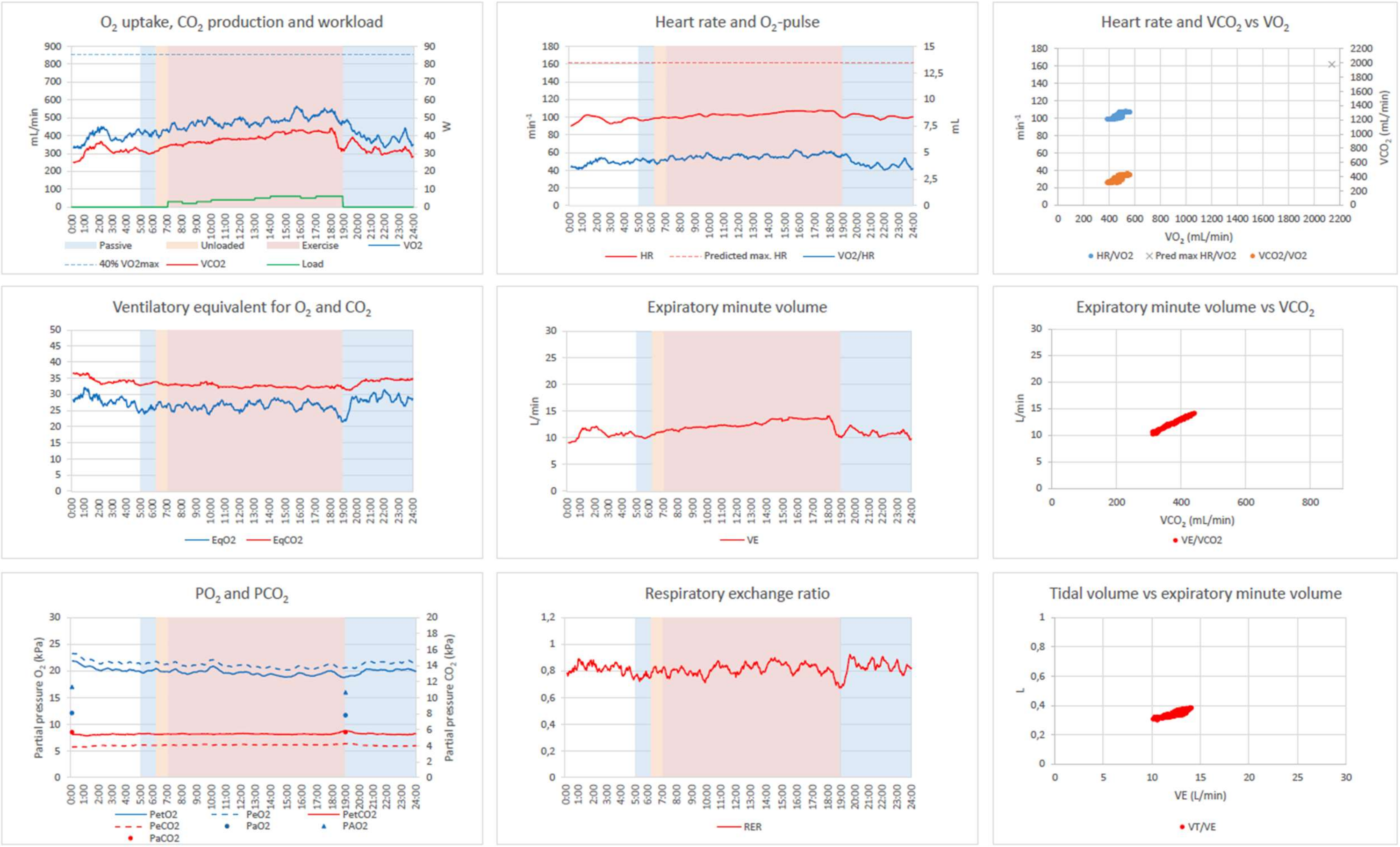

Patient #4
Additional plots ventilation

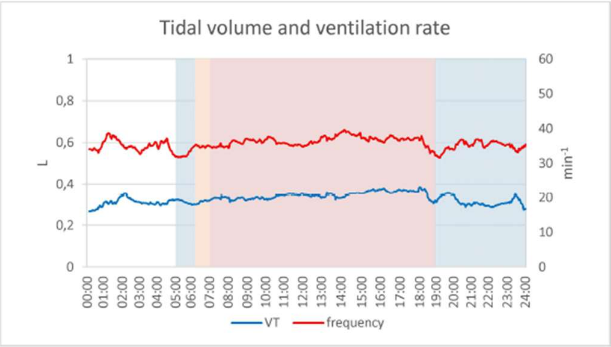

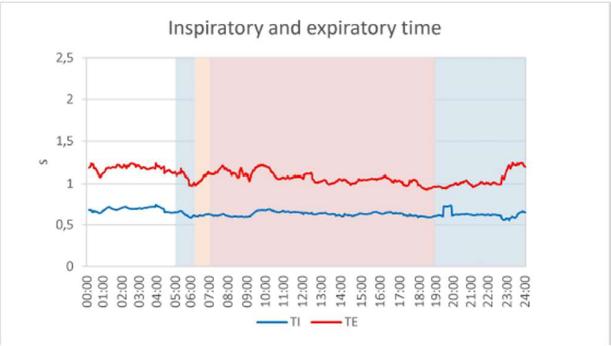

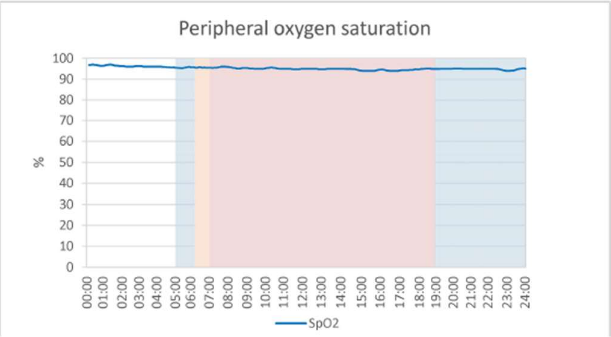

Additional plots anaerobic threshold

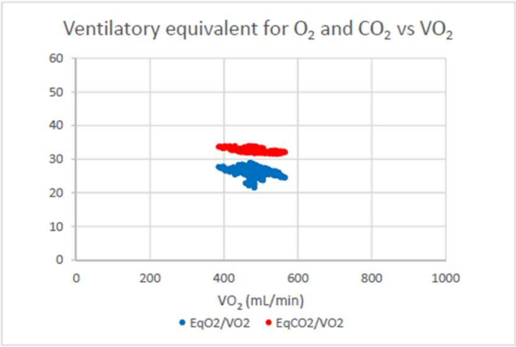

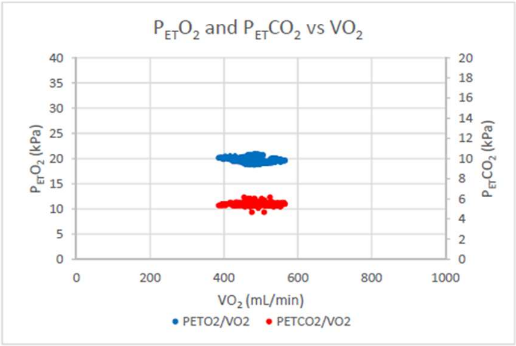

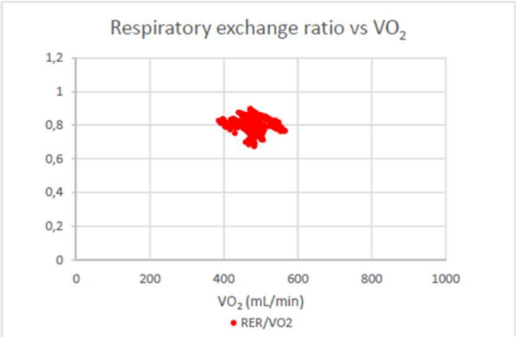

Patient 4

Ergometer: MOTOmed
Age: 58
Sex: male
Height (cm): 168
Weight (kg): 80
BMI (kg/m²): 28.3
Relevant comorbidity: none
Heart rhythm: sinus rhythm
Relevant medication: none

Admission diagnosis: Pancreatitis
Admission day (days): 29
Intubation day (days): 27
Airway: tracheostomy (size 6)
Ventilation mode: Pressure support ventilation
Pressure support (cm H_2O): 16
PEEP (cm H_2O): 8
F_iO_2 (%): 25

Work and metabolism

- Predicted $\dot{V}O_{2max}$: 2139 mL/min
- $\dot{V}O_2$ at rest: 391 mL/min – 4.89 mL/kg/min (high) – 18.3% of predicted $\dot{V}O_{2max}$
- Maximal workload (W): 6
- $\dot{V}O_{2peak}$: 565 mL/min – 7.06 mL/kg/min (low) – 26.4% of predicted $\dot{V}O_{2max}$
- MET: 1.45
- O_2 consumption increased slowly during unloaded exercise and reached a low level
- Increase in O_2 consumption in proportion to workload during exercise was normal
- Anaerobic threshold was not reached graphically
- RER at $\dot{V}O_{2peak}$: 0.77 (low)
- Lactate changed from 0.6 mmol/L (within normal range) before exercise to 0.7 mmol/L directly after exercise (within normal range)

Circulation

- HR at rest: 97 bpm
- Predicted maximum HR: 162 bpm
- Maximum HR: 108 bpm
- HRR: 54 bpm
- Maximum HR correlated with $\dot{V}O_{2peak}$
- Predicted maximum O_2-pulse: 13.2 mL/heartbeat
- O_2-pulse: 5.28 mL/heartbeat at peak exercise (low) – 40.0% of predicted O_2-pulse
- Blood pressure had a normal course during exercise
- ECG remained unchanged

Ventilation

- Maximal ventilation: 14.1 L/min (at $\dot{V}O_{2peak}$: 13.7 L/min)
- Maximal voluntary volume: unknown
- Breathing reserve: unknown
- Maximal tidal volume: 0.39 L (at $\dot{V}O_{2peak}$: 0.37 L)

Gas exchange

- P_aO_2 at rest was 12.1 kPa (within normal range) and had a slightly lower value of 11.7 kPa directly after exercise (within normal range)
- P_aCO_2 at rest was 5.7 kPa (within normal range) and had a similar value of 5.7 kPa directly after exercise
- Alveolar-arterial PO_2 gradient at rest was 5.09 kPa (elevated) and had a lower value of 4.44 kPa directly after exercise (within normal range)
- $P_{E(T)}O_2$ and $P_{E(T)}CO_2$ remained unchanged during exercise
- V_D/V_T at rest was 0.30 (within normal range) and 0.25 (within normal range) directly after exercise
- $EqCO_2$ at $\dot{V}O_{2peak}$ was 32.1 (within normal range)
- $P_{(a-ET)}CO_2$ at rest was 0.31 kPa (within normal range) and had a lower value of -0.13 kPa directly after exercise (within normal range)
- $P_{(ET-E)}CO_2$ at rest was 1.42 kPa and had a higher value of 1.58 kPa directly after exercise (within normal range)

Conclusion:

Basal metabolism was elevated (which is common after severe inflammation). Oxygen consumption increased normally during unloaded and loaded cycling, reaching a peak oxygen consumption of 26.4% of predicted, which was severely reduced. The anaerobic threshold was not reached. The relation between work and O_2 consumption appeared normal.
Heart rate increased normally in proportion to oxygen consumption, and the oxygen pulse showed a normal pattern, which suggested there was no circulatory limitation.
The A-a gradient of oxygen was normal after exercise, which complicates the interpretation of the slightly elevated value at rest. However, all indexes connected to dead space ventilation were normal, suggesting normal ventilation perfusion matching. During exercise, dead space decreased slightly, which was a normal response.
In summary, exercise tolerance was reduced, but the absence of circulatory and respiratory limitations suggested a restriction elsewhere in the musculoskeletal system.

Patient #5
Wasserman 9-panel plots

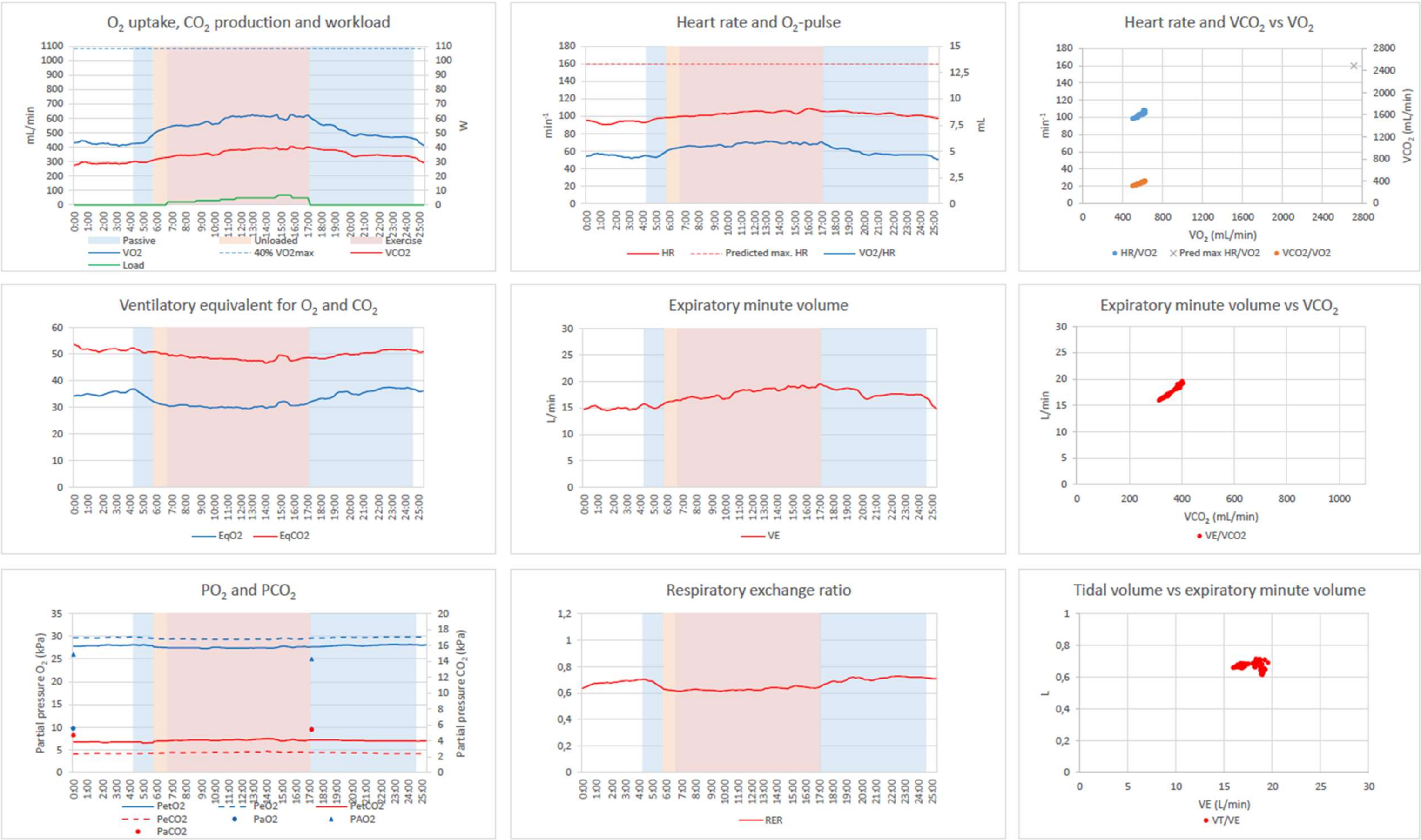

Patient #5
Additional plots ventilation

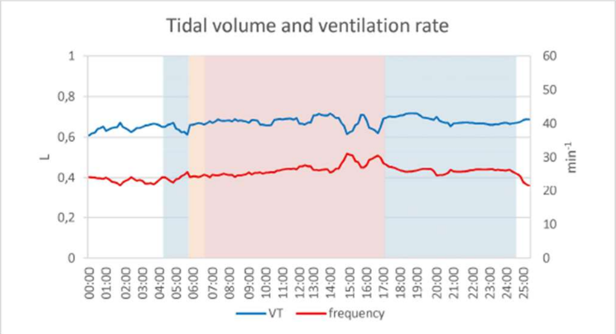

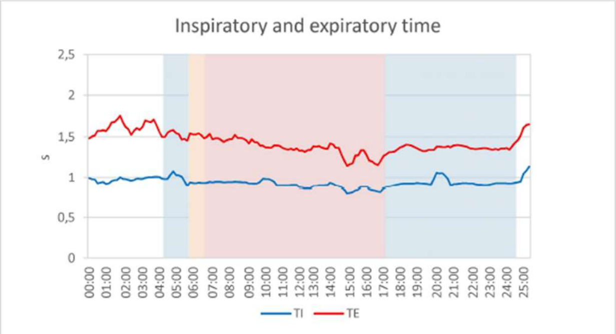

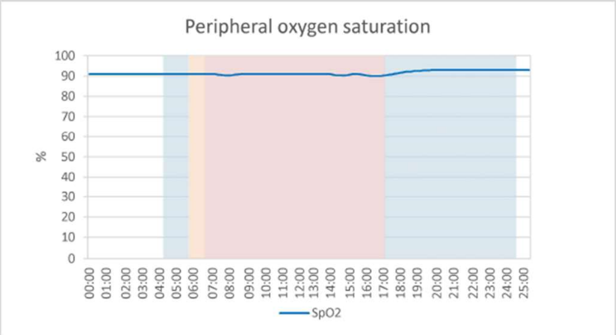

Additional plots anaerobic threshold

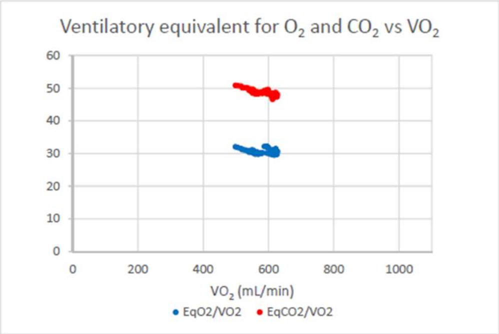

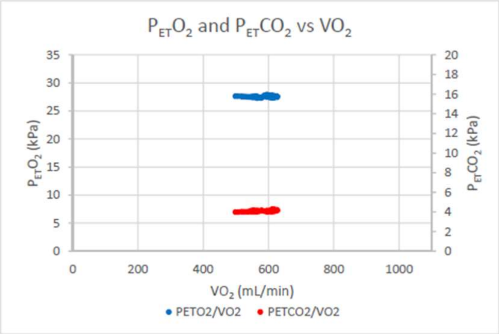

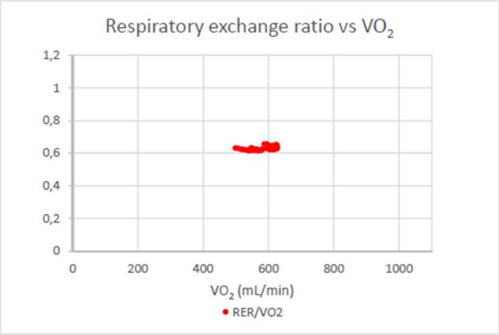

Patient 5

Ergometer: MOTOmed
Age: 60
Sex: male
Height (cm): 190
Weight (kg): 115
BMI (kg/m²): 31.6
Relevant comorbidity: ARDS (moderate)
Heart rhythm: atrial fibrillation
Relevant medication: none

Admission diagnosis: Pneumosepsis
Admission day (days): 15
Intubation day (days): 14
Airway: tracheostomy (size 6)
Ventilation mode: Pressure support ventilation
Pressure support (cm H_2O): 4
PEEP (cm H_2O): 8
F_iO_2 (%): 35

Work and metabolism
- Predicted $\dot{V}O_{2max}$: 2713 mL/min
- $\dot{V}O_2$ at rest: 424 mL/min – 3.69 mL/kg/min (high limit of normal) – 15.6% of predicted $\dot{V}O_{2max}$
- Maximal workload (W): 7
- $\dot{V}O_{2peak}$: 627 mL/min – 5.45 mL/kg/min (low) – 23.1% of predicted $\dot{V}O_{2max}$
- MET: 1.48
- O_2 consumption increased normally during unloaded exercise and reached a normal level
- Increase in O_2 consumption in proportion to workload during exercise was normal.
- Anaerobic threshold was not reached graphically
- RER at $\dot{V}O_{2peak}$: 0.63 (low)
- Lactate had similar value of 0.9 mmol/L (within normal range) before and directly after exercise

Circulation
- HR at rest: 93 bpm
- Predicted maximum HR: 160 bpm
- Maximum HR: 109 bpm
- HRR: 51 bpm
- HR/ $\dot{V}O_2$-slope was steep; possibly due to atrial fibrillation
- Predicted maximum O_2-pulse: 17.0 mL/heartbeat
- O_2-pulse: 5.93 mL/heartbeat at peak exercise (low) – 34.9% of predicted O_2-pulse
- Blood pressure had a normal course during exercise
- ECG remained unchanged

Ventilation
- Maximal ventilation: 19.6 L/min (at $\dot{V}O_{2peak}$: 19.2 L/min)
- Maximal voluntary volume: unknown
- Breathing reserve: unknown
- Maximal tidal volume: 0.72 L (at $\dot{V}O_{2peak}$: 0.65 L)

Gas exchange
- P_aO_2 at rest was 9.7 kPa (within normal range) and had a similar value of 9.5 kPa directly after exercise
- P_aCO_2 at rest was 4.7 kPa (within normal range) and had a higher value of 5.4 kPa directly after exercise (within normal range)
- Alveolar-arterial PO_2 gradient at rest was 17.1 kPa and had a lower value of 16.1 kPa directly after exercise (above normal range)
- $P_{E(T)}O_2$ and $P_{E(T)}CO_2$ remained unchanged during exercise
- V_D/V_T at rest was 0.49 (above normal range) and increased to 0.53 during exercise (far above normal range)
- $EqCO_2$ at $\dot{V}O_{2peak}$ was 48.1 (above normal range)
- $P_{(a-ET)}CO_2$ at rest was 0.84 kPa (above normal range) and had a higher value of 1.27 kPa directly after exercise (above normal range)
- $P_{(ET-E)}CO_2$ at rest was 1.46 kPa and had a higher value of 1.60 kPa directly after exercise (within normal range)

Conclusion:

Though at the upper limit of normal, basal metabolism was probably elevated, considering the patient's high BMI. Oxygen consumption increased normally during unloaded and loaded cycling, reaching a peak oxygen consumption of 23.1% of predicted, which indicated a severely reduced exercise tolerance. The anaerobic threshold was not reached. The relation between workload and O_2 consumption looked normal.
Heart rate increased normally in proportion to oxygen consumption, and the oxygen pulse showed a normal pattern, which suggested there was no circulatory limitation.
The A-a gradient of oxygen was very high, suggesting a diffusion problem or significant shunting of the pulmonary circulation. An elevated dead space fraction supported poor ventilation perfusion matching. During exercise, dead space increased, which was an abnormal response, confirmed by a concomitant increase in arterial-endtidal PCO_2 difference and a high ventilatory equivalent for CO_2. This could be consistent with pulmonary artery hypertension. The reduction of the ventilatory equivalent for CO_2 during exercise suggested improved ventilatory efficiency, but this was mainly achieved by an increase in arterial PCO_2, not by better ventilation.
In summary, the exercise tolerance was reduced. The result suggested that impaired gas exchange was a factor that limited exercise, pulmonary hypertension should be considered.

Patient #6
Wasserman 9-panel plots

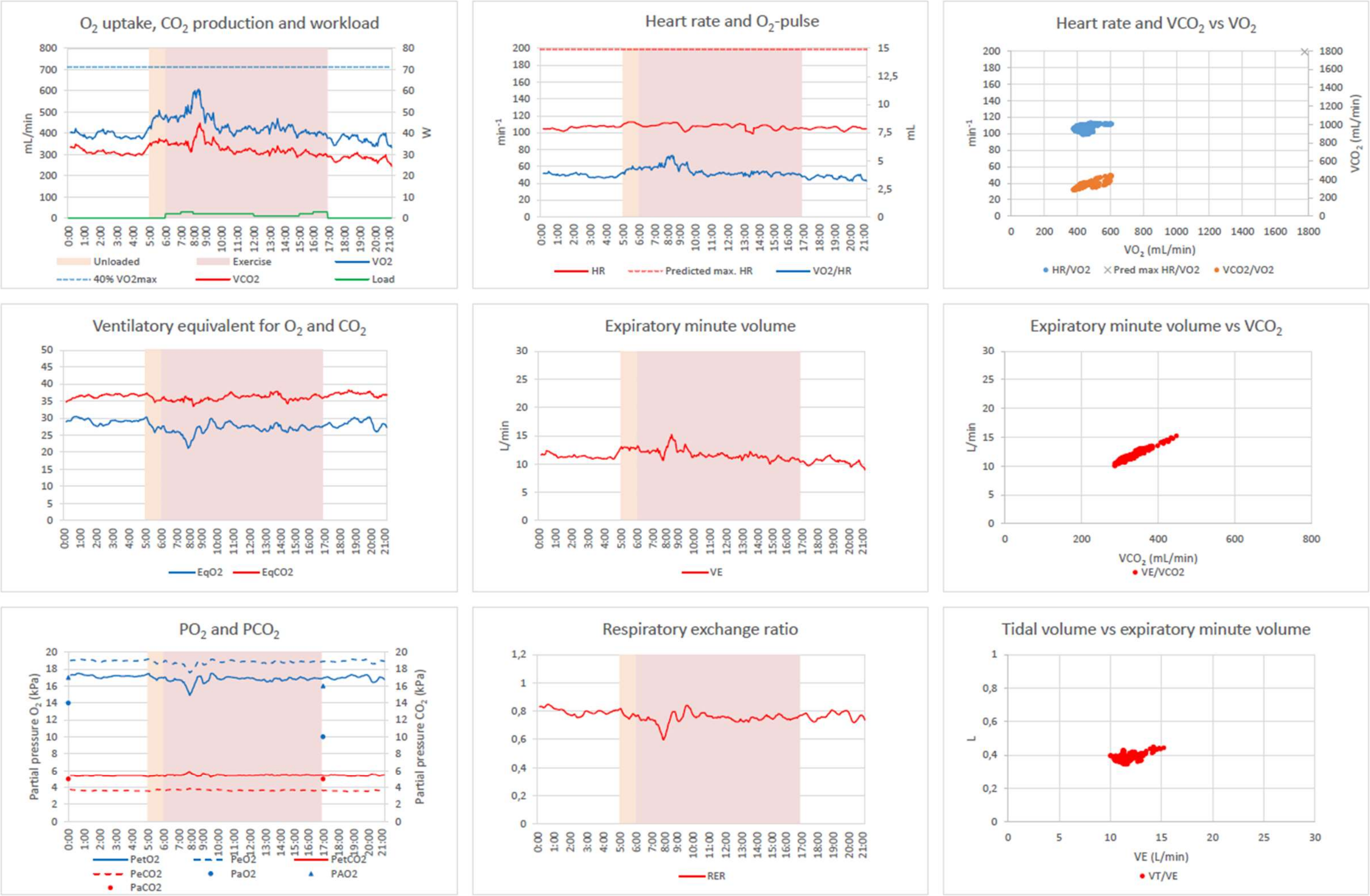

Patient #6
Additional plots ventilation

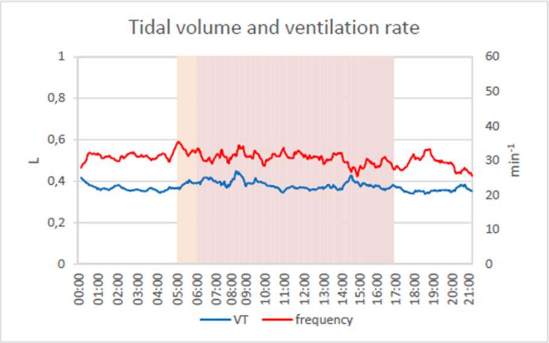

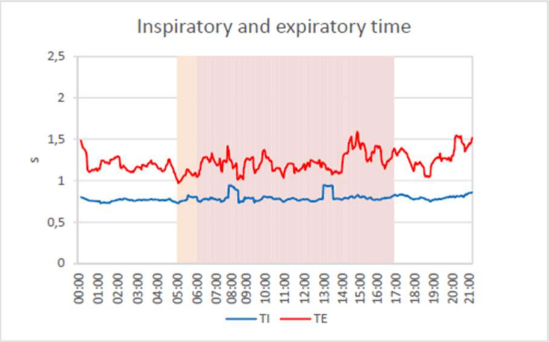

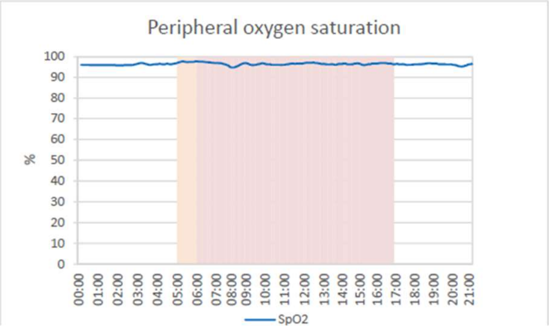

Additional plots anaerobic threshold

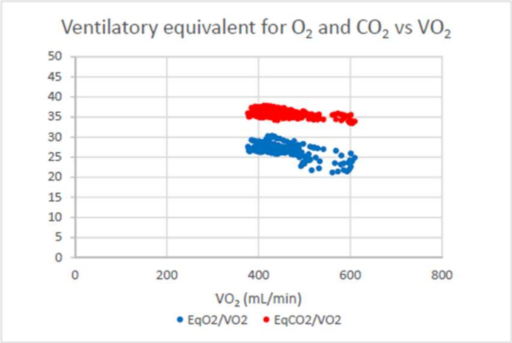

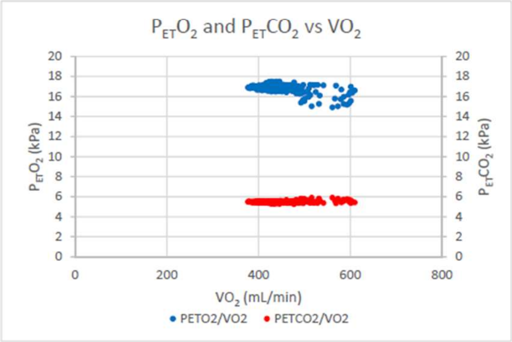

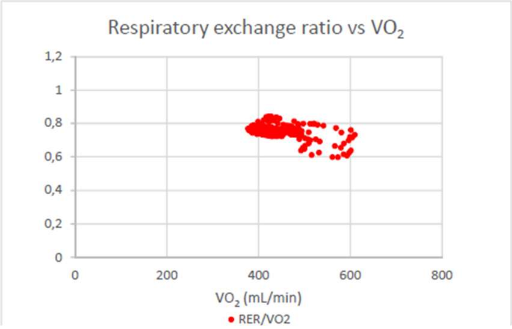

Patient 6

Ergometer: MOTOmed
Age: 21
Sex: female
Height (cm): 157
Weight (kg): 55
BMI (kg/m^2): 22.3
Relevant comorbidity: ARDS (severe)
Heart rhythm: sinus rhythm
Relevant medication: none

Admission diagnosis: Postanoxic encephalopathy
Admission day (days): 7
Intubation day: 7
Airway: tube (size 7)
Ventilation mode: Pressure support ventilation
Pressure support (cm H_2O): 11
PEEP (cm H_2O): 8
F_iO_2 (%): 35

Work and metabolism
- Predicted $\dot{V}O_{2max}$: 1777 mL/min
- $\dot{V}O_2$ at rest: 392 mL/min – 7.13 mL/kg/min (high) – 22.1% of predicted $\dot{V}O_{2max}$
- Maximal workload (W): 3
- $\dot{V}O_{2peak}$: 609 mL/min – 11.1 mL/kg/min (low) – 34.3% of predicted $\dot{V}O_{2max}$
- MET: 1.55
- O_2 consumption increased normally during unloaded exercise and reached a low level
- Increase in O_2 consumption in proportion to workload during exercise was not assessable
- Anaerobic threshold was not reached graphically
- RER at $\dot{V}O_{2peak}$: 0.73 (low)
- Lactate changed from 1.3 mmol/L (within normal range) before exercise to 1.2 mmol/L directly after exercise (within normal range)

Circulation
- HR at rest: 106 bpm
- Predicted maximum HR: 199 bpm
- Maximum HR: 112 bpm
- HRR: 87 bpm
- Maximum HR correlated with $\dot{V}O_{2peak}$
- Predicted maximum O_2-pulse: 8.9 mL/heartbeat
- O_2-pulse: 5.47 mL/heartbeat at peak exercise (low) – 61.3% of predicted O_2-pulse
- Blood pressure had a normal course during exercise
- ECG remained unchanged

Ventilation
- Maximal ventilation: 15.2 L/min (at $\dot{V}O_{2peak}$: 14.8 L/min)
- Maximal voluntary volume: unknown
- Breathing reserve: unknown
- Maximal tidal volume: 0.45 L (at $\dot{V}O_{2peak}$: 0.44 L)

Gas exchange
- P_aO_2 at rest was 14.1 kPa (above normal range) and had a lower value of 10.9 kPa directly after exercise (within normal range)
- P_aCO_2 at rest was 5.0 kPa (within normal range) and had a higher value of 5.6 kPa directly after exercise (within normal range)
- Alveolar-arterial PO_2 gradient at rest was 3.13 kPa and had a higher value of 5.40 kPa directly after exercise (above normal range)
- $P_{E(T)}O_2$ and $P_{E(T)}CO_2$ remained unchanged during exercise
- V_D/V_T at rest was 0.27 (within normal range) and had a higher value of 0.36 directly after exercise (above normal range)
- $EqCO_2$ at $\dot{V}O_{2peak}$ was 33.9 (within normal range)
- $P_{(a-ET)}CO_2$ at rest was -0.41 kPa (within normal range) and had a higher value of 0.12 kPa directly after exercise (within normal range)
- $P_{(ET-E)}CO_2$ at rest was 1.78 kPa and had a similar value of 1.81 kPa directly after exercise (above normal range)

Conclusion:

Basal metabolism was elevated (which is common after sepsis with ARDS). Oxygen consumption increased normally during unloaded cycling. During loaded cycling, peak oxygen consumption of 26.4% of predicted was reached at an unexpected moment, which might be due to mental restlessness, unrelated to external workload. The anaerobic threshold was not reached. The relation between work and oxygen consumption was unclear.
Heart rate did not increase during exercise, and was not blocked by medication, suggesting that the level of exercise was not challenging. The oxygen pulse showed a normal pattern during loaded and unloaded cycling, which suggested that at this level of exercise there was no circulatory limitation.
The arterial PO_2 was in the normal range, but this could be seen as abnormal at an FiO_2 of 35%. The A-a gradient of oxygen was at the upper limit of normal before exercise, and slightly above it afterwards, which should be considered abnormal at the age of 21. All indexes connected to dead space ventilation increased during exercise, which would be abnormal, but is hard to interpret in the absence of an increase in ventilatory minute volume.
In summary: Though there remained an impairment in oxygenation secondary to ARDS, the results did not suggest circulatory or respiratory limitations. Therefore, a restriction elsewhere in the musculoskeletal system, or absent motivation to exercise due to delirium seemed likely.

Patient #7
Wasserman 9-panel plots

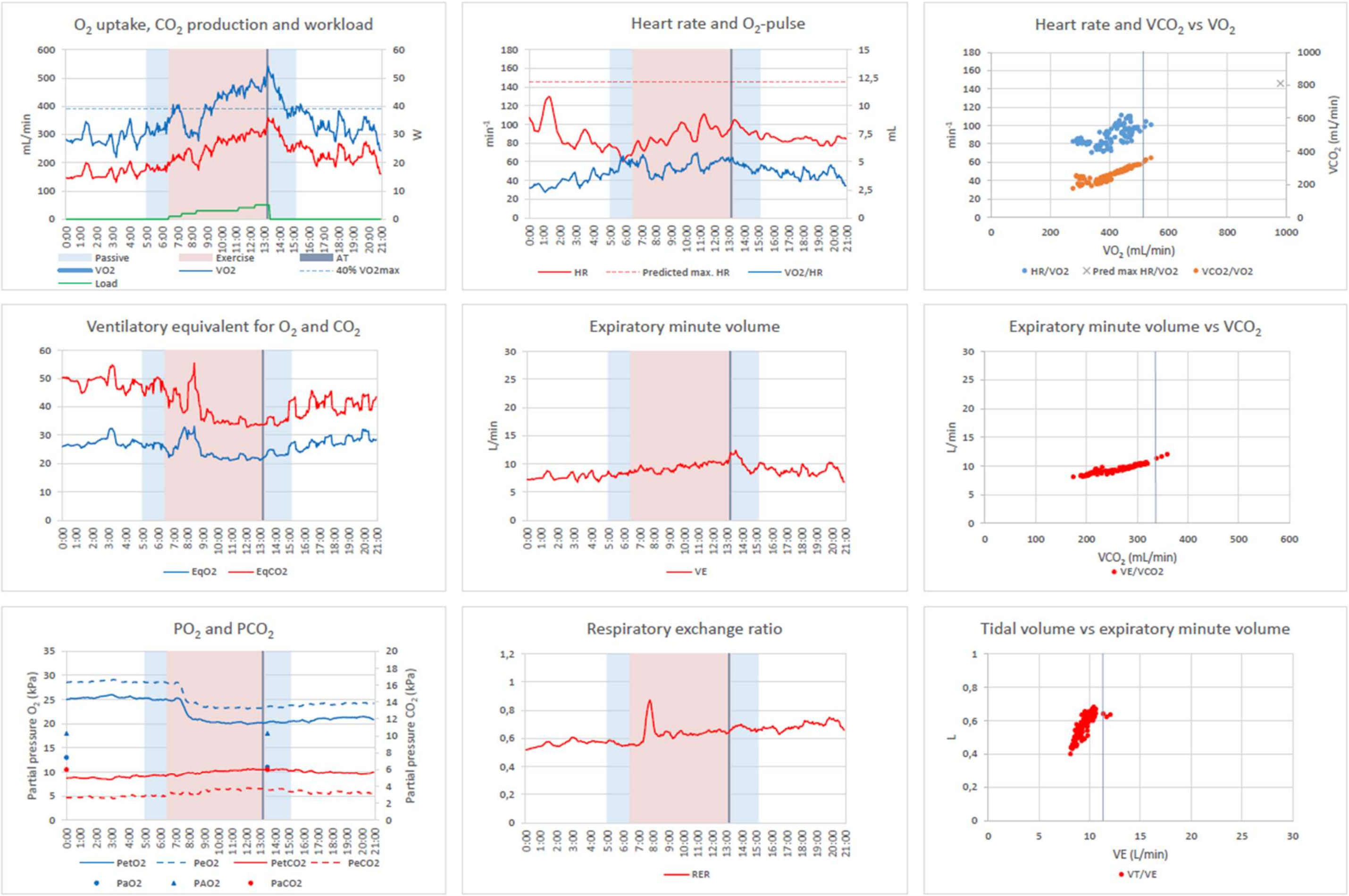

Patient #7
Additional plots ventilation

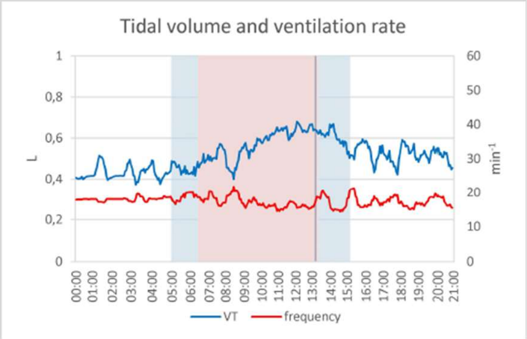

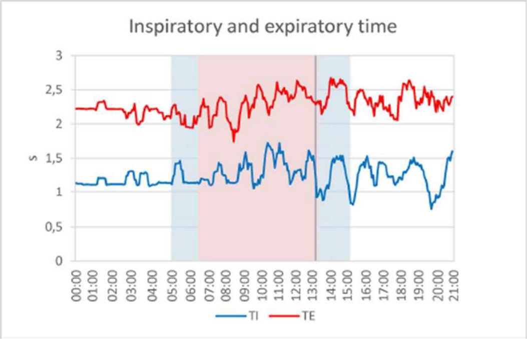

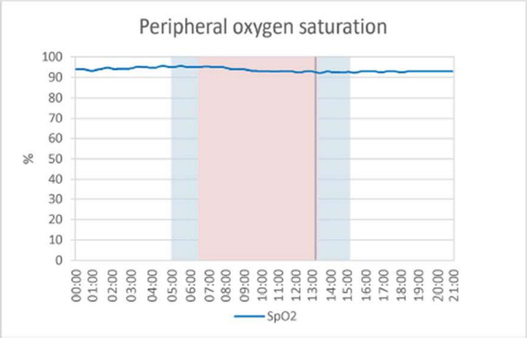

Additional plots anaerobic threshold

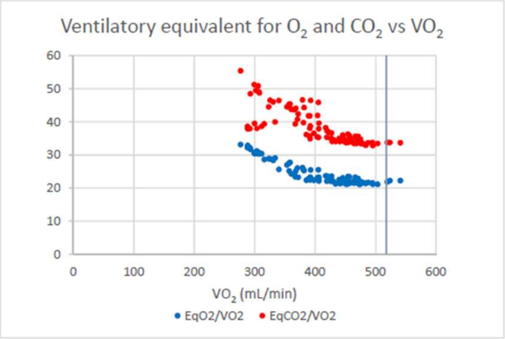

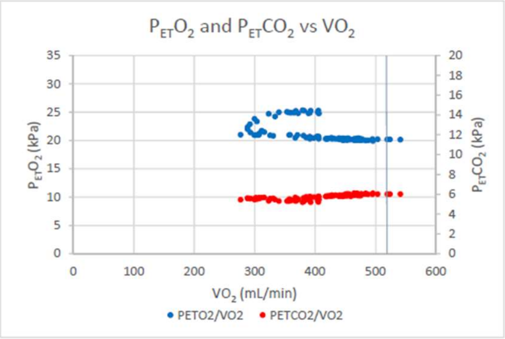

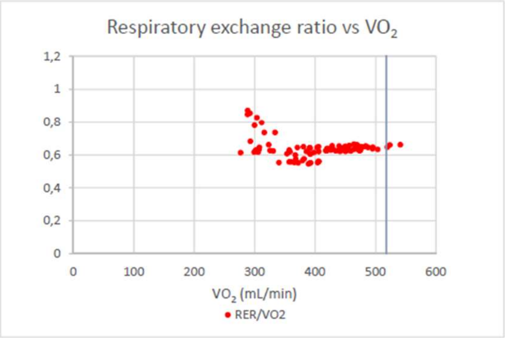

Patient 7

Ergometer: MOTOmed
Age: 74
Sex: female
Height (cm): 150
Weight (kg): 52
BMI (kg/m^2): 23.1
Relevant comorbidity: COPD (GOLD II) and severe ARDS
Heart rhythm: atrial fibrillation
Relevant medication: norepinephrine and digoxin

Admission diagnosis: Pneumosepsis
Admission day (days): 3
Intubation day: 2
Airway: tube (size 7)
Ventilation mode: Pressure regulated volume controlled ventilation (TV 400 mL)
Pressure (cm H_2O): 0
PEEP (cm H_2O): 8
F_iO_2 (%): 35
Spirometry: not available

Work and metabolism
- Predicted $\dot{V}O_{2max}$: 980 mL/min
- $\dot{V}O_2$ at rest: 287 mL/min – 5.52 mL/kg/min (high) – 29.3% of predicted $\dot{V}O_{2max}$
- Maximal workload (W): 5
- $\dot{V}O_{2peak}$: 541 mL/min – 10.4 mL/kg/min (low) – 55.2% of predicted $\dot{V}O_{2max}$
- MET: 1.89
- Unloaded cycling was omitted from the protocol; O_2 consumption increased normally during exercise
- Increase in O_2 consumption in proportion to workload during exercise was high
- A mild increase in RER during loaded exercise and a subtle increase in EqO_2 at the end of exercise suggest that the anaerobic threshold was reached at a $\dot{V}O_2$ of 500 mL/min, corresponding to to 51.0% of predicted $\dot{V}O_{2max}$
- RER at $\dot{V}O_{2peak}$: 0.66 (low)
- Lactate changed from 0.8 mmol/L (within normal range) before exercise to 1.0 mmol/L directly after exercise (within normal range)

Circulation
- HR at rest: 91 bpm
- Predicted maximum HR: 146 bpm
- Maximum HR: 104 bpm
- HRR: 42 bpm
- Maximum HR correlated with $\dot{V}O_{2peak}$
- Predicted maximum O_2-pulse: 6.7 mL/heartbeat
- O_2-pulse: 5.39 mL/heartbeat at peak exercise (normal) – 80.2% of predicted O_2-pulse
- Blood pressure had a normal course during exercise
- ECG remained unchanged

Ventilation
- Patient was at a volume controlled ventilation mode, limiting the possibilities to increase minute ventilation.
- Minute ventilation increased from 7.5 L/min to 12.0 L/min at $\dot{V}O_{2peak}$
- Maximal voluntary volume: unknown
- Breathing reserve: unknown
- Tidal volume increased from 0.4 L to 0.68 L (at $\dot{V}O_{2peak}$: 0.64 L)

Gas exchange
- After approximately one minutes of loaded exercise, F_iO_2 was reduced from 35% to 30% causing an artefact in the $\dot{V}O_2$ curve
- P_aO_2 at rest was 13.2 kPa (within normal range) and had a lower value of 11.5 kPa directly after exercise (within normal range)
- P_aCO_2 at rest was 6.3 kPa (above normal range) and had a slightly higher value of 6.8 kPa directly after exercise
- Alveolar-arterial PO_2 gradient at rest was 9.9 kPa and had a lower value of 7.2 kPa directly after exercise (above normal range; even with correction for an altered/lower F_iO_2)
- $P_{E(T)}O_2$ abruptly decreased during exercise due to a reduction of F_iO_2. $P_{E(T)}CO_2$ increased during exercise
- V_D/V_T at rest was 0.57 (above normal range) and had a lower value of 0.43 directly after exercise (above normal range)
- $EqCO_2$ at $\dot{V}O_{2peak}$ was 33.6 (within normal range)
- $P_{(a-ET)}CO_2$ at rest was 1.29 kPa and had a lower value of 0.78 kPa directly after exercise (above normal range)
- $P_{(ET-E)}CO_2$ at rest was 2.29 kPa and had a similar value of 2.30 kPa directly after exercise (above normal range)

Conclusion:

Basal metabolism was elevated (which is common after sepsis). Oxygen consumption increased normally during loaded cycling, reaching a peak oxygen consumption of 55.2% of predicted (which was relatively good for a critically ill patient). The oxygen consumption was high in proportion to workload (this may be seen in obese patients, which this patient certainly was not). There was some suggestion that the anaerobic threshold was reached at an oxygen consumption of 500 ml/min, although this was not convincing. Serum lactate at the end of exercise had not increased. Heart rate and oxygen pulse increased normally in proportion to oxygen consumption. As there was still heart rate reserve at the end of exercise, the limitation to exercise was not circulatory. The volume controlled ventilation mode still allowed the patient to adjust the minute volume to requirement; the tidal volume increased by 70% during exercise. Respiratory rate did not increase, suggesting that ventilation was not the limiting factor.
The A-a gradient of oxygen was high, suggesting a diffusion problem or significant shunting of the pulmonary circulation. An elevated dead space fraction supported poor ventilation perfusion matching. During exercise, dead space decreased by much, which was also reflected by improved ventilatory efficiency and a reduction in arterial-endtidal PCO_2 difference, but it remained high, even at peak exercise. The large difference between endtidal and mixed expiratory PCO_2 is suggestive of COPD.
In summary, exercise tolerance was reduced. The results suggested impaired gas exchange as a causative factor, contributed to by poor matching of perfusion to ventilation secondary to ARDS on a background of COPD.
.
